# Supplementary material for: Randomised double-blind phase 3 clinical study testing impact of atorvastatin on prostate cancer progression after initiation of androgen deprivation therapy: study protocol
Source: BMJ Open. 2022 Apr 29;12(4):e050264. doi: 10.1136/bmjopen-2021-050264 (PMC9058683; doi:10.1136/bmjopen-2021-050264)
Supplement: Author's manuscript [file bmjopen-2021-050264.draft_revisions.pdf]

BMJ Open is committed to open peer review. As part of this commitment we make the peer review history of every article we publish publicly available.

When an article is published we post the peer reviewers' comments and the authors' responses online. We also post the versions of the paper that were used during peer review. These are the versions that the peer review comments apply to.

The versions of the paper that follow are the versions that were submitted during the peer review process. They are not the versions of record or the final published versions. They should not be cited or distributed as the published version of this manuscript.

BMJ Open is an open access journal and the full, final, typeset and author-corrected version of record of the manuscript is available on our site with no access controls, subscription charges or pay-per-view fees (http://bmjopen.bmj.com).

If you have any questions on BMJ Open's open peer review process please email info.bmjopen@bmj.com

BMJ Open

Randomized double-blind phase 3 clinical study testing impact of atorvastatin on prostate cancer progression after initiation of androgen deprivation therapy - study protocol

Journal:	*BMJ Open*
Manuscript ID	bmjopen-2021-050264
Article Type:	Protocol
Date Submitted by the Author:	16-Feb-2021
Complete List of Authors:	Siltari, Aino; Tampere University, Faculty of Medicine and Health Technology; University of Helsinki, Faculty of Medicine, Pharmacology<br>Riikonen, Jarno; Tampere University Hospital, Department of Urology<br>Koskimäki, Juha; Tampere University Hospital, Department of Urology<br>Pakarainen, Tomi; Tampere University Hospital, Department of Urology<br>Ettala, Otto; University of Turku<br>Boström, Peter; University of Turku<br>Seikkula, Heikki; Central Finland Central Hospital<br>Kotsar, Andres; Tartu University Hospital<br>Tammela, Teuvo; University of Tampere<br>Helminen, Mika; Tampere University, Health Sciences<br>Lehtimäki, Terho; Tampere University, Department of Clinical Chemistry<br>Fode, Mikkel; Herlev Hospital<br>Østergren, Peter; Herlev and Gentofte University Hospital, Department of Urology<br>Borre, Michael; Aarhus Universitetshospital, Department of Urology<br>Rannikko, Antti; Helsinki University and Helsinki University Hospital, Department of Urology<br>Marttila, Timo; Seinäjoki Central Hospital, Department of Urology<br>Salonen, Arto; Kuopio University Hospital<br>Ronkainen, Hanna; Oulu University Hospital<br>Murtola, Teemu J ; Tampere University Hospital, Department of Urlogy
Keywords:	Prostate disease < UROLOGY, Clinical trials < THERAPEUTICS, Urological tumours < ONCOLOGY

SCHOLARONE™
Manuscripts

Randomized double-blind phase 3 clinical study testing impact of atorvastatin on prostate cancer progression after initiation of androgen deprivation therapy - study protocol

Aino Siltari[1], Jarno Riikonen[2], Juha Koskimäki[2], Tomi Pakarainen[2], Otto Ettala[3], Peter Boström[3], Heikki Seikkula[4], Andres Kotsar[5], Teuvo LJ Tammela[1,2], Mika Helminen[7,8], Terho Lehtimäki[1,9], Mikkel Fode[10], Peter Østergren[10], Michael Borre[11], Antti Rannikko[12], Timo Marttila[13], Arto Salonen[14], Hanna Ronkainen[15], Teemu J Murtola[1,2*]

[1]Tampere University, Faculty of Medicine and Health Technology, Tampere, Finland, [2]TAYS Cancer Center, Department of Urology, Tampere, Finland, [3]Turku University Hospital and University of Turku, Turku, Finland, [4]Central Finland Central Hospital, Jyväskylä, Finland, [5]Tartu University Hospital, Department of Urology, Tartu, Estonia, [6]Fimlab laboratories, Department of Pathology, Tampere, Finland, [7]Tampere University Hospital, Research Services, Tampere, Finland, [8]Tampere University, Faculty of Social Sciences, Health Sciences, Tampere Finland, [9]Fimlab Laboratories, Department of Clinical Chemistry, Tampere, Finland, [10]Department of Urology, Herlev and Gentofte Hospital, Denmark, [11]Aarhus University Hospital, Department of Urology, Aarhus, Denmark, [12]Helsinki University and Helsinki University Hospital, Department of Urology, Helsinki, Finland [13]Seinäjoki Central Hospital, Department of Urology, Seinäjoki, Finland, [14]Kuopio University Hospital, Department of Urology, Kuopio, Finland, [15]Oulu University Hospital, Department of Urology, Oulu, Finland

*corresponding author: Teemu J Murtola, Department of Urology, Tampere University Hospital, PL 2000, 33521 Tampere, Finland, teemu.murtola@tuni.fi, telephone: +358 (0) 294 52 11.

Keywords: Prostate cancer; castration-resistant prostate cancer; androgen deprivation therapy; atorvastin; randomized clinical trial

Word count: 3604 (excluding title page, references, figures, and tables)

For peer review only

Abstract

Introduction

Blood cholesterol is likely a risk factor for prostate cancer prognosis and use of statins is associated with lowered risk of prostate cancer recurrence and progression. Furthermore, use of statins has been associated with prolonged time before development of castration resistance (CR) during androgen deprivation therapy (ADT) for prostate cancer. However, the efficacy of statins on delaying castration-resistance has not been tested in a randomized placebo-controlled setting.

This study aims to test statins' efficacy compared to placebo in delaying development of CR during ADT treatment for primary metastatic or recurrent prostate cancer in a randomized, placebo-controlled, double-blind clinical trial. Secondary aim is to explore effect of statin intervention on prostate cancer mortality and lipid metabolism during ADT.

Methods and analysis

A total of 400 men with *de novo* metastatic prostate cancer or recurrent disease after primary treatment and starting ADT will be recruited and randomized 1:1 to use daily 80 mg of atorvastatin or placebo. Patients are followed until disease recurrence or death. Primary outcome is time to formation of CR after initiation of ADT. Serum lipid levels are analyzed to test whether changes in serum cholesterol parameters during ADT predict length of treatment response. Futhermore, the trial will compare quality of life, cardiovascular morbidity, changes in blood glucose and circulating cell-free DNA, and urine lipidome during trial.

Ethics and dissemination

This study is approved by the Regional ethics committees of the Pirkanmaa Hospital District, Science center, Tampere, Finland (R18065M) and Tarto University Hospital, Tarto, Estonia (319/T-6). All participants read and sign informed consent form before study entry. Clinicaltrial.gov: NCT04026230, Eudra-CT: 2016-004774-17, protocol code: ESTO2, protocol date September 10th 2020 and version 6.

Keywords: Prostate cancer; castration-resistant prostate cancer; androgen deprivation therapy; atorvastin; randomized clinical trial

Strengths and limitations of this study

For peer review only

- First randomized placebo-controlled phase 3 clinical study on effects of atorvastatin on prostate cancer progression during ADT treatment.
- Clarifies the role of lipid metabolism in prostate cancer progression
- Investigates whether management of most severe cases of prostate cancer could be improved using cheap well-tolerated drug with established cardiovascular benefits
- Investigates the effects of atorvastatin treatment on glucose tolerance, mortality, and quality of life
- As a limiting factor, only minority of prostate cancer cases are advanced and large proportion of potential participants are ineligible due to using statins already. Thus, study enrolment will take several years

Introduction

Prostate cancer (PCa) is the most common cancer in Finnish men and a major public health burden causing annually around 900 cancer deaths in Finland and the yearly costs caused by the disease are estimated to reach 180 million euros.[1-2] However, not all prostatic malignancies are lethal; only 10-20% of tumors advance to metastases and eventually into a fatal stage. Advanced PCa is treated by androgen deprivation therapy (ADT). Eventually, however, PCa progress despite the ADT treatment and forms state called castration resistance. Thus, castration resistance is clinically defined as a moment when prostate cancer no longer responds to ADT treatment. Median time to castration resistance is 12-15 months and 45 months to death in patients who have started ADT for metastatic prostate cancer.[3-7] In men who start ADT for disease recurrence after primary treatment, median failure-free survival time is 33 months and 70 months to death.[3]

Blood cholesterol is likely a risk factor for prostate cancer prognosis; risk of disease recurrence after primary treatment is significantly elevated in men with hypercholesterolemia.[8] Importance of cholesterol is also supported by studies demonstrating effects of cholesterol on prostate cancer cell growth.[9,10] Furthermore, upregulation of intracellular lipid production and ensuing lipid accumulation is essential in surviving hypoxic tumor microenvironment.[11] In prostate cancer cells it also assists in evading host-tumor immune response.[12] Also, upregulation of intracellular cholesterol production appears to be central for development of castration resistance.[13]

For peer review only - http://bmjopen.bmj.com/site/about/guidelines.xhtml

Lipid metabolism is emerging as a new risk factor for prostate cancer progression. The role of cholesterol is important in development of castration resistance, and inhibition of intracellular lipid production interferes with androgen receptor (AR) signaling essential for prostate cancer progression[13-15]. Cholesterol is precursor for intracellular androgen production, a central mechanism for prostate cancer cells to overcome ADT.[13,15] A recent study suggests that some of the serum basic lipid parameters may have increased association with development of castration resistance and metastasis especially in statin-naïve patients.[16] Clarification of the role of lipid metabolism during ADT will likely provide new tools for control of disease progression and prostate cancer treatment.

Use of cholesterol-lowering statin drugs is associated with lowered risk of prostate cancer recurrence and progression; risk of prostate cancer death is reduced by 30% compared to the non-users.[14,17,18] The anticancer effect may be specifically against progression of the disease and the mechanisms driving it.[19] Statins inhibit the cholesterol-synthesizing mevalonate pathway, which is active in prostate cancer cells.[10,20] Besides cholesterol, this pathway produces also isoprenoid proteins which are critical for regulation of cell growth and other central cellular control processes.[20] Furthermore, steroid hormones such as testosterone are metabolized from cholesterol, thus statins appear to target androgen metabolism, another crucial pathway for prostate cancer growth.[21,22]

Statin use has been reported to prolong efficacy of ADT in prostate cancer as it has been reported to prolong the efficacy of ADT for 8-10 months.[23,24] Furthermore, statins have been linked to a prolonged response to androgen-signaling targeted drugs abiraterone and enzalutamide used in management of castration-resistant disease.[25,26]

Statins have not been found to affect prostate cancer mortality in trials testing their efficacy in secondary prevention of cardiovascular disease.[27] However, in these trials cancer was often an exclusion criterion. On the other hand, it may be that statin treatment has more impact on hormone-dependent cancers which are underrepresented in these studies. In a randomized clinical trial focusing on prostate cancer patients 80 mg of atorvastatin has been found to reduce tumor proliferation activity compared to placebo after minimum exposure of 21 days.[28] Similar results were seen after treatment with fluvastatin, although in a non-randomized and uncontrolled setting.[29] Nevertheless, clinical efficacy of statins in preventing progression of prostate cancer has not been tested in a randomized placebo-controlled setting. Also, a recent post-hoc study of randomized clinical trial concluded that statin use was associated with decreased overall and PCa-specific mortality in men with ADT.[30] Therefore, it is important to do a trial testing effects of statins specifically in prostate cancer patients.

For peer review only

Study objective

Primary objective for this phase III randomized double-blind placebo-controlled trial is to explore whether intervention with atorvastatin delays prostate cancer progression i.e. development of castration resistance compared to placebo during androgen deprivation therapy (ADT) for metastatic or recurrent prostate cancer. Secondary objectives include exploring whether atorvastatin lowers prostate cancer-specific or overall mortality compared to placebo, and to demonstrate whether changes in serum lipid parameters predict disease recurrence and occurrence of adverse tumor genomic traits predicting castration resistance among prostate cancer patients during ADT.

Methods and analysis

Study setting

Study flow, study settings, and other information are presented in Figure 1 and Table 1. The study recruitment target is 400 participants who start ADT as primary management of *de novo* metastatic prostate cancer or as secondary management for prostate cancer recurrence after localize treatment. Participants can be high-risk M0 or M1 stage, main inclusion criterion is that long-term ADT treatment is started. These men will be randomized 1:1 (200 + 200) to receive either 80 mg of atorvastatin daily or placebo until disease recurrence i.e. development of castration resistance, death, or maximum of ten years. Sample size is based on a power calculation from a previous retrospective study.[23]

The study will be carried out in collaboration between urological departments of University Hospitals and central hospitals in Finland, the Herlev University Hospital in Denmark, and the Tartu University Hospital in Estonia (Table 1).

Study data were collected and managed using REDCap electronic data capture tools hosted at Tampere University.[31,32] Only the primary investigators, study nurses, and registered study co-investigator from each participating site will have access to the platform. All laboratory results, symptoms, and results from imaging studies done at the discretion of the attending clinicians are recorded in the database. All clinical decisions besides the study drug, e.g. use of early chemotherapy, abiraterone, or other drugs in adjunct with ADT will be up to the discretion of the attending clinician and allowed but will also be recorded in the trial database.

Follow-up is continued until development of castration resistance, death, or maximum of 10 years. Participants are given the opportunity to carry on with the intervention even after development of

For peer review only

castration resistance to observe effects on survival. Unblinding will be performed after recruitment target has been reached and all participants have been followed for minimum of 12 months.

Castration resistance is defined as either PSA progression (three consecutive rises of PSA measured at least 1 week apart with two > 50% increases over the nadir and PSA > 2 ng/ml) or radiological progression (appearance of two or more lesions in bone scan or soft tissue enlargement as per RECIST criteria) while serum testosterone is at the castrate level (< 50 ng/ml or 1.7 nmol/l).

For men who initiate statin use during the study period for clinical indications, the study drug is dropped but the study follow-up is continued. These men will be included in the final analysis according to the intention-to-treat principle within their allocated study arm.

In case of intolerable side-effects as judged by either the participant or the attending physician, the study drug is stopped, and these men will be analyzed according to the intention-to-treat principle.

Participants who discontinue follow-up or deviate from the study protocol for any reason will be given the chance to remain or return to follow-up to allow intention-to-treat analyses.

Inclusion and exclusion criteria

Inclusion criteria for participants are histopathologically confirmed metastatic adenocarcinoma of the prostate or high-risk M0 stage recurrent prostate cancer for which androgen deprivation or antiandrogen therapy is initiated no longer than 3 months before recruitment, willingness to participate, and signing of informed consent (Figure 1).

Exclusion criteria for participants are regular statin use at the time of recruitment or within 6 months of it, previous adverse effects during statin therapy, familial hypercholesterolemia or very high total cholesterol (9.3 mmol/l or above), clinically significant renal (serum creatinine above 170 µmol/l) or liver insufficiency (serum alanine aminotransferase more than two times above the upper limit of normal range), and use of drugs that may interact with statins (St John's Wort, HIV protease inhibitors, ciclosporin, macrolide antibiotics, fucidic acid, phenytoin, carbamazepine, dronedarone, or oral antifungal medication) (Figure 1).

Study endpoints

Primary endpoint is the time to disease progression after starting ADT/antiandrogen therapy. Secondary endpoints are 1); prostate cancer-specific mortality and overall survival, 2); change in

For peer review only

serum cholesterol during the intervention and its role in predicting time to disease recurrence in the placebo arm, 3); occurrence of adverse tumor traits predicting development of castration resistance in circulating cell free DNA, 4); changes in fasting blood glucose during ADT, 5); occurrence of cardiovascular events during ADT, and 6); Quality of life (QoL) during ADT.

Study flow

Study flow and schedule is presented in Figure 1 and Table 2. Patients are recruited at urology outpatient clinic at urologists' visits. If the inclusion criteria are met and the exclusion criteria are not and the participant signs informed consent form, he is given a study number (ranging between E-001 to E-400) and he receives either 80 mg atorvastatin or placebo according to the study arm randomly allocated for the study number. No blocking or other restrictions will be implemented for randomization. Only the national study coordinator in each country will be able to see which study arm the participant has been randomized to. The participant will then receive first dose of the respective study drug randomized to his study number. The drug boxes and capsules containing atorvastatin and placebo will be of identical outlook. All researchers, nurses, and study participants will remain blinded to the allocation sequence until termination of the study and closure of the study data.

Control visits are scheduled at 6-month intervals to suit common clinical practice. Control visits are done by a clinician, complemented with measurements done by a research nurse. For each control visit the participant returns remaining capsules from the previously delivered ration and will receive a new one according to his study number. Number of the remaining capsules will be counted and saved in the trial database to monitor compliance. In every visit serum PSA, alkaline phosphatase, creatinine, and fasting glucose will be measured (Table 2) and the participant is asked about symptoms that are suggestive of metastases and about possible adverse effects of the study drug. Serum creatine kinase is measured at the baseline.

Blood samples are taken and stored at 6-month intervals to monitor changes in tumor markers and occurrence of adverse tumor characteristics after initiation of ADT. Quality of life during the intervention will be charted once a year using validated WHOQOL-BREF quality of life questionnaire.[33]

Follow-up is continued until development of castration resistance, drop out from the study, death, or maximum of ten years. Participants developing castration resistance are given an opportunity to carry on with their assigned treatment as statins have been linked to longer cancer-specific survival.

For peer review only

Unblinding will be performed after recruitment target has been reached and all participants have been followed for minimum of 12 months.

Prostate cancer treatment apart from the study drug will be up to the discretion of the attending clinician. All laboratory results, symptoms, and possible imaging results will be recorded in the trial database. All clinical decisions regarding early chemotherapy and imaging will be made on the discretion of the clinician and recorded in the trial database.

Stored blood samples

Separate whole blood (for RNA and DNA isolation), plasma, and serum samples are taken at each control visit at 6-month intervals and stored for mass-spectrometric and nuclear magnetic resonance (NMR)-based determination of serum lipidome. These samples will also be used for RNA and whole genome sequencing of mutations and genetic modifications predicting disease recurrence and metastasis, such as BRCA1/2, ERG, MYC, TP53, ATM, PTEN, and AR splice variants.[34,35] Also hypoxia markers will be measured.[36]

For participants with confirmed metastases in the bone or soft tissues, amount of cell free DNA from the plasma will be measured before ADT initiation and again at castration resistance development for sequencing and detection of genetic modifications predicting metastases.[34,35] Selected patients are also imaged with positron emission tomography (PET) scan using 18F-2-nitroimidazolpentafluoropropylacetamide (EF5)[37] to monitor hypoxia and FDG-PET to evaluate immune responses[38] in the primary tumor and the metastases during the atorvastatin intervention.

Power calculations

In a cohort study by Harshman et al.[23], among men starting ADT, 58% of the statin users and 75% of non-users of statins progress to castration resistance during median 5.8 years. We used these crude percentages for sample size calculations.

With alfa and beta values of 0.05 and 0.20 (power=0.80) the difference of 58% *vs*. 75% would require 132 + 132 observations, if group sizes are assumed to be similar. The program “PS - Power and sample size, version 3.1.2” was used for the calculation. To take possible dropouts into account we set the sample size to 400 men, 200 in each study arm.

The median time to disease progression is assumed to be 12-15 months for patients with *de novo* metastatic disease and 33 months for patients recurring after primary therapy.[3-7] Therefore, the

For peer review only

intervention will continue until castration resistance, death, or for maximum of 10 years. Post hoc follow-up will continue after the intervention.

It has to be noted that the number of studies published on this topic is low and, in the absence of randomized evidence on this topic, the power calculation is based on results of epidemiological study prone to residual bias. Therefore, an interim analysis will be performed after the first 100 participants have met the primary endpoint, i.e. progressed into castration resistance. Without lifting the blinding, the power calculations are repeated to verify whether the chosen sample size has adequate statistical power to detect statistically significant difference of the effect size observed between the randomization groups at that time. If the calculations suggest that a larger sample size is needed for the trial, the study protocol will be amended with updated recruitment target.

Statistical methods

Distribution balance of prostate cancer clinical characteristics such as TNM stage at diagnosis, Gleason score, and PSA at time of diagnosis will be compared between the study arms using chi-square test for categorical variables and Mann-Whitney U-test, Student's t-test, or ANOVA for continuous variables depending on whether they follow normal distribution.

Recurrence-free time, i.e. time from ADT initiation to castration resistance, will be compared between the study arms using Kaplan-Meier curves and log-rank test. Risk of death, as well as risk for occurrence of adverse tumor traits among men receiving atorvastatin compared to men receiving placebo will be estimated using Cox proportional hazards regression.

Statistical significance of the association between changes in serum lipid levels and occurrence of adverse tumor genomic traits will be estimated using ANOVA or Mann-Whitney U-test depending on the distribution normality.

Subgroup analyses will be performed stratified by type of ADT or antiandrogen therapy initially selected; GnRH agonists/antagonists, antiandrogen, orchiectomy, or other. Men who start with antiandrogen therapy often change to GnRH agonists/antagonists or orchiectomy when PSA increases during ADT. This is allowed and PSA recurrence during antiandrogen therapy is not considered as disease progression as serum testosterone needs to be at castrate level as well for meeting the primary endpoint.

Another subgroup analysis will be stratified by whether or not the participants have received local therapy (radiation or surgery) for prostate cancer in addition to ADT and by type of additional therapy

For peer review only

participant has received; docetaxel, abiraterone, enzalutamide, or apalutamide at hormone-sensitive stage in addition to ADT.

All analyses will include only participants with available data, imputations will not be used.

Ethics and dissemination

This study is approved by the regional ethics committee of Pirkanmaa Hospital District, Science center, Tampere, Finland (R18065M). All participants read and sign informed consent form before study entry. The results of this study will be published in international peer reviewed journals.

The trial is ethical for the following reasons 1); The study aims to improve treatment of metastatic prostate cancer, which is the second most common cause of cancer death in Western countries, 2); the potential scientific and societal benefits from the project are substantial. If the study hypothesis proves to be right, it will provide an entirely new way to prevent and/or delay progression of metastatic prostate cancer with atorvastatin, a drug that is cheap, well-tolerated, and with established cardiovascular benefits, 3); the study drug (atorvastatin) is known to be well-tolerated and it is currently widely used in management of hypercholesterolemia and cardiovascular disease. The adverse effects caused by the drug are usually mild and transient after stopping the study drug, and 4); randomized, placebo-controlled design ensures the study will produce highest quality evidence that will change prostate cancer treatment guidelines if the hypothesis proves to be right.

After publication of results for the primary endpoints, anonymized summary metadata and statistical code will be made openly available. The data will not include any information that could make it possible to identify a given participant.

Quality control

The study steering committee includes investigators from each participating recruitment center. The steering committee will meet twice a year to oversee trial progression of the recruitment, integrity of collected data and discuss possible protocol amendments.

External trained study monitors independent from the study sponsors ensure the data quality and good clinical practice by making regular yearly check-up visits to participating study centers. This will be separately arranged in each participating country.

Reporting and registering of adverse effects

For peer review only

The participants are advised to contact the researchers in case of suspected adverse effect related to the study drug. If the side-effects are intolerable the study drug will be discontinued. In uncertain cases serum levels of creatine kinase, alanine aminotranspherase and creatinine are checked to see if any of these have changed considerably compared to the baseline. If the laboratory tests are not normal, or if the participant or the attending physician finds the side-effects alarming the study drug will be discontinued. Study database records specifically most common adverse effects muscular pain, elevated fasting blood glucose (> 6,1 mmol/l) and elevated serum creatine kinase (>280 U/l), even when they do not lead to discontinuation of the study drug.

Trial database includes separate question for serious adverse effects as evaluated by CTCAE criteria, version 5.0. If serious side-effects are reported, trial database asks to fulfill separate query where detailed description of the adverse effect is given according to CTCAE criteria. Study sponsor and coordinator will be automatically notified via e-mail if serious adverse effect is reported in the database. All serious adverse effect that threatens life or health are reported to national authority (FIMEA in Finland) within 7 days of detection. If a life-threatening adverse effect is suspected blinding will be lifted for the participant in case to see whether he has received atorvastatin or placebo. Unexpected adverse effects will be reported to national authority within 15 days of detection.

Patients and public involvement

In the design phase of the trial, primary investigator presented the study protocol in numerous public events, including patient advocacy group meetings, giving opportunity for the audience to give feedback and suggestions how to improve the study. Patient advocacy organizations of prostate cancer patients are actively involved in finding subjects for the study through announcements in their journals and by allowing investigators frequently promote the study in their meetings. Study results will be reported also in patient organization journals and social media sites for maximum visibility and distribution of trial outcomes.

References

1. Finnish cancer registry statistics: http://stats.cancerregistry.fi/stats/eng/veng0003m0.html
2. Mäklin S, Rissanen P. Cancer expenses in Finland: http://www.cancer.fi/@Bin/47734/Syöpien+aiheuttamat+kustannukset.pdf
3. Ross RW Xie W, Regan MM, Pomerantz M, *et al*. Efficacy of androgen deprivation therapy (ADT) in patients with advanced prostate cancer: association between Gleason score,

For peer review only

prostate-specific antigen level, and prior ADT exposure with duration of ADT effect. *Cancer* 2008;**112**:1247-53. doi: 10.1002/cncr.23304.

4. Botrel TE, Clark O, Lima Pompeo AC, *et al*. Efficacy and Safety of Combined Androgen Deprivation Therapy (ADT) and Docetaxel Compared with ADT Alone for Metastatic Hormone-Naive Prostate Cancer: A Systematic Review and Meta-Analysis. *PLoS One* 2016;**11**:e0157660. doi: 10.1371/journal.pone.0157660.
5. Sweeney CJ, Chen YH, Carducci M, *et al.* Chemohormonal Therapy in Metastatic Hormone-Sensitive Prostate Cancer. *N Engl J Med* 2015;**373**:737-46. doi: 10.1056/NEJMoa1503747
6. Seo WI, Kang PM, Kim TH, *et al*. Primary androgen deprivation therapy for prostate cancer in koreans: a retrospective multicenter study. World J Mens Health. 2014;32(3):159-66.
7. Mottet N, Van Damme J, Loulidi S, Russel C, Leitenberger A, Wolff JM. Intermittent hormonal therapy in the treatment of metastatic prostate cancer: a randomized trial. *BJU Int* 2012;**110**:1262-9. doi: 10.1111/j.1464-410X.2012.11120.x.
8. Arthur R, Møller H, Garmo H, *et al*. Association between baseline serum glucose, triglycerides and total cholesterol, and prostate cancer risk categories. *Cancer Med* 2016;**5**:1307-18. doi: 10.1002/cam4.665
9. Murtola TJ, Syvälä H, Pennanen P, *et al*. Comparative effects of high and low-dose simvastatin on prostate epithelial cells: the role of LDL. *Eur J Pharmacol* 2011;**673**:96-100. doi: 10.1016/j.ejphar.2011.10.022.
10. Murtola TJ, Syvälä H, Pennanen P, *et al.* The importance of LDL and cholesterol metabolism for prostate epithelial cell growth. *PLoS One* 2012;**7**:e39445. doi: 10.1371/journal.pone.0039445.
11. Ackerman D, Simon MC. Hypoxia, lipids, and cancer: surviving the harsh tumor microenvironment. *Trends Cell Biol* 2014;**24**:472-8. doi: 10.1016/j.tcb.2014.06.001.
12. Herber DL, Cao W, Nefedova Y *et al*. Lipid accumulation and dendritic cell dysfunction in cancer. *Nat Med* 2010;**16**:880-6. doi: 10.1038/nm.2172
13. Han W, Gao S, Barrett D, *et al*. Reactivation of androgen receptor-regulated lipid biosynthesis drives the progression of castration-resistant prostate cancer. *Oncogene* 2018;**37**:710-21. doi: 10.1038/onc.2017.385.
14. Raval AD, Thakker D, Negi H, *et al.* Association between statins and clinical outcomes among men with prostate cancer: a systematic review and meta-analysis. *Prostate Cancer Prostatic Dis* 2016;**19**:222. doi: 10.1038/pcan.2016.3

For peer review only

15. Barfeld SJ, Itkonen HM, Urbanucci A, *et a*l. Androgen-regulated metabolism and biosynthesis in prostate cancer. *Endocr Relat Cancer* 2014;**21**:T57-66. doi: 10.1530/ERC-13-0515.
16. Dambal S, Howard LE, Allott EH, *et al*. Serum Lipids prior to Starting Androgen Deprivation Therapy and Risk of Castration Resistant Prostate Cancer and Metastasis: Results from the SEARCH Database. *J Urol* 2020;**203**:120-7. doi: 10.1097/JU.0000000000000494
17. Van Rompay MI, Solomon KR, Nickel JC, *et al*. Prostate cancer incidence and mortality among men using statins and non-statin lipid-lowering medications. *Eur J Cancer* 2019;**112**:118-26. doi: 10.1016/j.ejca.2018.11.033.
18. Kumar A, Riviere P, Luterstein E, *et al*. Associations among statins, preventive care, and prostate cancer mortality. *Prostate Cancer Prostatic Dis* 2020. doi: 10.1038/s41391-020-0207-5
19. Murai T. Cholesterol lowering: role in cancer prevention and treatment. *Biol Chem* 2015;**396**:1-11. doi: 10.1515/hsz-2014-0194.
20. Garcia-Ruiz C, Morales A, Fernandez-Checa JC. Statins and protein prenylation in cancer cell biology and therapy. *Anticancer Agents Med Chem* 2012;**12**:303-15. doi: 10.2174/187152012800228715
21. Armandari I, Hamid AR, Verhaegh G, *et al*. Intratumoral steroidogenesis in castration-resistant prostate cancer: a target for therapy. *Prostate Int* 2014;**2**:105-13. doi: 10.12954/PI.14063.
22. Mokarram P, Alizadeh J, Razban V, *et al*. Interconnection of Estrogen/Testosterone Metabolism and Mevalonate Pathway in Breast and Prostate Cancers. *Curr Mol Pharmacol* 2017;**10**:86-114. doi: 10.2174/1874467209666160112125631.
23. Harshman LC, Wang X, Nakabayashi M, et al. Statin use at the time of initiation of androgen deprivation therapy and time to progression in patients with hormone-sensitive prostate cancer. *JAMA Oncol* 2015;**1**:495-504. doi: 10.1001/jamaoncol.2015.0829.
24. Jung J, Lee C, Lee C, et al. Effects of statin use on the response duration to androgen deprivation therapy in metastatic prostate cancer. *Korean J Uro*l 2015;**56**:630-6. doi: 10.4111/kju.2015.56.9.630.
25. Syvälä H, Pennanen P, Bläuer M, *et al*. Additive inhibitory effects of simvastatin and enzalutamide on androgen-sensitive LNCaP and VCaP prostate cancer cells. *Biochem Biophys Res Commun* 2016;**481**:46-50. doi: 10.1016/j.bbrc.2016.11.021

For peer review only

26. Yang H, Pang L, Hu X, *et al.* The effect of statins on advanced prostate cancer patients with androgen deprivation therapy or abiraterone/enzalutamide: A systematic review and meta-analysis. *J Clin Pharm Ther* 2020;**45**:488-95. doi: 10.1111/jcpt.13092
27. Lv HL, Jin DM, Liu M, *et al*. Long-term efficacy and safety of statin treatment beyond six years: a meta-analysis of randomized controlled trials with extended follow-up. *Pharmacol Res* 2014;**81**:64-73. doi: 10.1016/j.phrs.2014.02.006.
28. Murtola T, Riikonen J, Syvälä H, *et al.* Atorvastatin Versus Placebo for Prostate Cancer Before Radical Prostatectomy-A Randomized, Double-blind, Placebo-controlled Clinical Trial. *Eur Urol* 2018;**74**:697-701. doi: 10.1016/j.eururo.2018.06.037.
29. Longo J, Hamilton RJ, Masoomian M, *et al.* A pilot window-of-opportunity study of preoperative fluvastatin in localized prostate cancer. *Prostate Cancer Prostatic Dis* 2020 ;**23**:630-37. doi: 10.1038/s41391-020-0221-7
30. Hamilton RJ, Ding K, Crook JM, *et al.* The Association Between Statin Use and Outcomes in Patients Initiating Androgen Deprivation Therapy. *Eur Urol* 2020 (article in press) https://doi.org/10.1016/j.eururo.2020.12.031
31. Harris PA, Taylor R, Thielke R, *et al.* Research electronic data capture (REDCap) – A metadata-driven methodology and workflow process for providing translational research informatics support. *J Biomed Inform* 2009;**42**:377-81. doi: 10.1016/j.jbi.2008.08.010
32. Harris PA, Taylor R, Minor BL, *et al*. The REDCap consortium: Building an international community of software partners. *J Biomed Inform* 2019;**95**:103208. doi: 10.1016/j.jbi.2019.103208.
33. WHOQOL-BREF, The World Health Organization Quality of Life, THL
34. Mateo J, Boysen G, Barbieri CE, *et al*. DNA repair in prostate cancer: biology and clinical implications. *Eur Urol* 2017;**71**:417-25. doi: 10.1016/j.eururo.2016.08.037
35. Xu J, Qiu Y. Role of androgen receptor splice variants in prostate cancer metastasis. *Asian J Urol* 2016;**3**:177-84. doi: 10.1016/j.ajur.2016.08.003.
36. Le QT, Courter D. Clinical biomarkers for hypoxia targeting. *Cancer Metastasis Rev* 2008;**27**:351-62. doi: 10.1007/s10555-008-9144-9
37. Stieb S, Eleftheriou A, Warnock G*, et al*. Longitudinal PET imaging of tumor hypoxia during the course of radiotherapy. *Eur J Nucl Med Mol Imaging* 2018;**45**:2201-17.doi: 10.1007/s00259-018-4116-y
38. Hunt SJ, Seraj SM, Alavi A. PET Assessment of Immune Effects from Interventional Oncology Procedures. *PET Clin* 2019;**14**:477-85. doi: 10.1016/j.cpet.2019.06.007.

For peer review only

Authors' contributions: Teemu Murtola is the primarily investigator of this study and designed the study with the help from other authors. Aino Siltari and Teemu Murtola wrote the first draft of the manuscript. All other authors further contributed to manuscript preparation and accepted the final version of the manuscript.

Funding: This study is supported by grants from Finnish Cancer Foundation (grant numbers 024194 and 3122800563 (TJM) and 16112016 (TL)), Nordic Cancer Union (grant number MS793 (TJM)), Päivikki and Sakari Sohlberg Foundation (grant number 31228005901 (AS)), competitive research grant of Pirkanmaa hospital district (grant number 9X032 (TJM) and grant X51001 (TL)), and the Academy of Finland (grants numbers 322098 (TL), and 3121330724 (TJM)).

Disclaimer: None of the funders has a role in the design, management, analysis or interpretation of data, or writing and publication of results.

Competing interests: Peter Østergren: honorarium as speaker from Ipsen A/S, Ferring Pharmaceuticals, and Astellas Pharma. Mikkel Fode: consultant fees and honorarium as speaker from Astellas and Ferring. Hanna Ronkainen: consultant fees from Bayer AB and honorarium as speaker from Sanofi. Teemu J Murtola: Consultant fees from Astellas, Janssen, speaker's honorarium from Astellas, Janssen and Sanofi, participation in scientific meetings at the expense of Ferring, Pfizer, and Sanofi, stockholder for Arocell AB. Peter Boström: consultant fees from Astellas Pharma. All other authors: No competing interests to declare.

Data sharing: There are no additional unpublished data at this point of this study.

Captions to Figure and Tables:

Figure 1. Study flow with inclusion and exclusion criteria.

Table 1. Study setting of Impact of atorvastatin on prostate cancer after initiation of androgen deprivation therapy -clinical trial

Table 2. Study flow and timing of the laboratory and blood sample collection

For peer review only

Table 1. Study setting of Impact of atorvastatin on prostate cancer after initiation of androgen deprivation therapy -	
**Study settings**	
Primary registry and trial identifying number	Clinicaltrials.gov NCT04026230
Date of registration in primary registry	July 19, 2019
Secondary identifying numbers	Eudra-CT: 2016-004774-17, Protocol code: ESTO2
Source(s) of monetary or material support	Tampere University Hospital, Finland
Primary sponsor	Tampere University Hospital, Finland
Secondary sponsor(s)	Helsinki University Hospital,Turku University Hospital, Central Finland Central Hospital, Kuopio University Hospital, Oulu University Hospital, Finland, Herlev Hospital, Denmark, University Hospital Tarto, Estonia
Contact for public queries	Tampere University Hospital, Teemu Murtola, MD, PhD
Contact for scientific queries	Tampere University Hospital, Teemu Murtola, MD, PhD
Public title	Impact of atorvastatin on prostate cancer progression after initiation of androgen deprivation therapy
Scientific title	Impact of atorvastatin on prostate cancer progression after initiation of androgen deprivation therapy – lipid metabolism as a novel biomarker to predict prostate cancer progression – phase 3, double-blind randomized clinical trial FinnProstata XV
Countries of recruitment	Finland, Denmark, Estonia
Health condition studied	Metastatic or recurrent prostate cancer
Intervention	Active comparator: Capsules of atorvastatin 80 mg, Placebo comparator: Similar capsules as in the atorvastatin arm, but without the active ingredient
Key inclusion and exclusion criteria	Inclusion criteria: Histopathologically confirmed metastatic adenocarcinoma of the prostate for which androgen deprivation or antiandrogen therapy is initiated no longer than 3 months before as the primary treatment<br>Willingness to participate and signing of informed consent<br>Exclusion criteria: Statin use at the time of recruitment or within 6 months of it, Previous adverse effects during statin therapy, familial hypercholesterolemia or very high total cholesterol ,clinically significant renal or liver insufficiency, use of drugs that may interact with statins
Sexes eligible for study:	Male
Accepts healthy volunteers	No
Study type	Interventional, Allocation: randomized, Intervention model: parallel assignment with 1:1 allocation ratio, Masking: double blind (subject, caregiver, investigator, outcomes assessor), Primary purpose: prevention, Phase III
Date of first enrolment	July 2019
Target sample size	400
Recruitment status	Recruiting
Primary outcome(s)	Castration resistance
Key secondary outcomes	Lipid levels, Prostate cancer mortality and overall survival, Circulating cell free DNA, Fasting blood glucose, Occurrence of cardiovascular events during ADT, Quality of life

For peer review only

Table 2. Study flow and timing of the laboratory and blood sample collection

	Enrollment	Allocation	Follow-up				
TIMEPOINT		0	6m	12m	18m	24m	etc*
ENROLLMENT:							
Eligibility screen	x						
Informed consent	x						
Allocation		x					
INTERVENTIONS:+							
Atorvastatin			x	x	x	x	
Placebo			x	x	x	x	
ASSESMENTS:							
Serum PSA			x	x	x	x	
Alkaline phosphatase			x	x	x	x	
Creatinine	x		x	x	x	x	
Serum creatine kinase	x						
Alanine aminotranspherase	x						
Fasting blood glucose	x		x	x	x	x	
Lipid levels	x		x	x	x	x	
Cell free DNA (subgroup)^	x						
QoL	x			x		x	

m=month, y=year, QoL=quality of life, PSA=prostate-specific antigen

*follow-up until disease recurrance, death, or max. 10 years

+ allocation 1:1 to have eighter 80 mg atorvastatin or placebo daily

^Cell free DNA is measured before allocation and after occurrence of castration resistance.

For peer review only

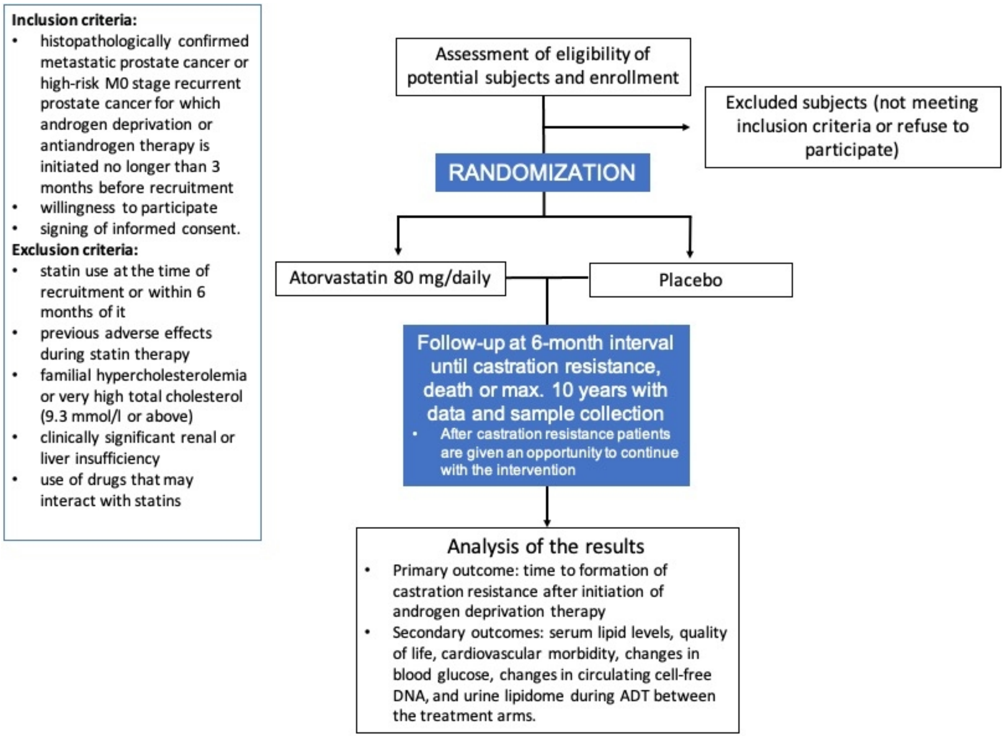

Study flow with inclusion and exclusion criteria.

284x208mm (72 x 72 DPI)

SPIRIT

STANDARD PROTOCOL ITEMS: RECOMMENDATIONS FOR INTERVENTIONAL TRIALS

SPIRIT 2013 Checklist: Recommended items to address in a clinical trial protocol and related documents*

Section/item	Item No	Description	Addressed on page number
**Administrative information**			
Title	1	Descriptive title identifying the study design, population, interventions, and, if applicable, trial acronym	1
Trial registration	2a	Trial identifier and registry name. If not yet registered, name of intended registry	2
	2b	All items from the World Health Organization Trial Registration Data Set	
Protocol version	3	Date and version identifier	2
Funding	4	Sources and types of financial, material, and other support	14
Roles and responsibilities	5a	Names, affiliations, and roles of protocol contributors	1
	5b	Name and contact information for the trial sponsor	1
	5c	Role of study sponsor and funders, if any, in study design; collection, management, analysis, and interpretation of data; writing of the report; and the decision to submit the report for publication, including whether they will have ultimate authority over any of these activities	14
	5d	Composition, roles, and responsibilities of the coordinating centre, steering committee, endpoint adjudication committee, data management team, and other individuals or groups overseeing the trial, if applicable (see Item 21a for data monitoring committee)	10

For peer review only

For peer review only - http://bmjopen.bmj.com/site/about/guidelines.xhtml

Introduction

Background and rationale	6a	Description of research question and justification for undertaking the trial, including summary of relevant studies (published and unpublished) examining benefits and harms for each intervention	___3-4_______
	6b	Explanation for choice of comparators	___3-4_________
Objectives	7	Specific objectives or hypotheses	____5_____
Trial design	8	Description of trial design including type of trial (eg, parallel group, crossover, factorial, single group), allocation ratio, and framework (eg, superiority, equivalence, noninferiority, exploratory)	___5-6_____

Methods: Participants, interventions, and outcomes

Study setting	9	Description of study settings (eg, community clinic, academic hospital) and list of countries where data will be collected. Reference to where list of study sites can be obtained	___5-6_________
Eligibility criteria	10	Inclusion and exclusion criteria for participants. If applicable, eligibility criteria for study centres and individuals who will perform the interventions (eg, surgeons, psychotherapists)	_____6_______
Interventions	11a	Interventions for each group with sufficient detail to allow replication, including how and when they will be administered	____7-8_______
	11b	Criteria for discontinuing or modifying allocated interventions for a given trial participant (eg, drug dose change in response to harms, participant request, or improving/worsening disease)	_______6______
	11c	Strategies to improve adherence to intervention protocols, and any procedures for monitoring adherence (eg, drug tablet return, laboratory tests)	____7-8________
	11d	Relevant concomitant care and interventions that are permitted or prohibited during the trial	______8______
Outcomes	12	Primary, secondary, and other outcomes, including the specific measurement variable (eg, systolic blood pressure), analysis metric (eg, change from baseline, final value, time to event), method of aggregation (eg, median, proportion), and time point for each outcome. Explanation of the clinical relevance of chosen efficacy and harm outcomes is strongly recommended	_______6-7____
Participant timeline	13	Time schedule of enrolment, interventions (including any run-ins and washouts), assessments, and visits for participants. A schematic diagram is highly recommended (see Figure)	_7-8,Table 2_____

Sample size	14	Estimated number of participants needed to achieve study objectives and how it was determined, including clinical and statistical assumptions supporting any sample size calculations	_____8-9______
Recruitment	15	Strategies for achieving adequate participant enrolment to reach target sample size	_____8-9_______
**Methods: Assignment of interventions (for controlled trials)**			
Allocation:			
Sequence generation	16a	Method of generating the allocation sequence (eg, computer-generated random numbers), and list of any factors for stratification. To reduce predictability of a random sequence, details of any planned restriction (eg, blocking) should be provided in a separate document that is unavailable to those who enrol participants or assign interventions	________7____
Allocation concealment mechanism	16b	Mechanism of implementing the allocation sequence (eg, central telephone; sequentially numbered, opaque, sealed envelopes), describing any steps to conceal the sequence until interventions are assigned	______7_____
Implementation	16c	Who will generate the allocation sequence, who will enrol participants, and who will assign participants to interventions	____7________
Blinding (masking)	17a	Who will be blinded after assignment to interventions (eg, trial participants, care providers, outcome assessors, data analysts), and how	______7_____
	17b	If blinded, circumstances under which unblinding is permissible, and procedure for revealing a participant's allocated intervention during the trial	_____7_______
**Methods: Data collection, management, and analysis**			
Data collection methods	18a	Plans for assessment and collection of outcome, baseline, and other trial data, including any related processes to promote data quality (eg, duplicate measurements, training of assessors) and a description of study instruments (eg, questionnaires, laboratory tests) along with their reliability and validity, if known. Reference to where data collection forms can be found, if not in the protocol	______7-8____
	18b	Plans to promote participant retention and complete follow-up, including list of any outcome data to be collected for participants who discontinue or deviate from intervention protocols	_____6______

Data management	19	Plans for data entry, coding, security, and storage, including any related processes to promote data quality (eg, double data entry; range checks for data values). Reference to where details of data management procedures can be found, if not in the protocol	________5_____
Statistical methods	20a	Statistical methods for analysing primary and secondary outcomes. Reference to where other details of the statistical analysis plan can be found, if not in the protocol	__9-10_________
	20b	Methods for any additional analyses (eg, subgroup and adjusted analyses)	_9-10_________
	20c	Definition of analysis population relating to protocol non-adherence (eg, as randomised analysis), and any statistical methods to handle missing data (eg, multiple imputation)	___10_______
**Methods: Monitoring**			
Data monitoring	21a	Composition of data monitoring committee (DMC); summary of its role and reporting structure; statement of whether it is independent from the sponsor and competing interests; and reference to where further details about its charter can be found, if not in the protocol. Alternatively, an explanation of why a DMC is not needed	_____10______
	21b	Description of any interim analyses and stopping guidelines, including who will have access to these interim results and make the final decision to terminate the trial	____6, 11______
Harms	22	Plans for collecting, assessing, reporting, and managing solicited and spontaneously reported adverse events and other unintended effects of trial interventions or trial conduct	______11_______
Auditing	23	Frequency and procedures for auditing trial conduct, if any, and whether the process will be independent from investigators and the sponsor	______10______
**Ethics and dissemination**			
Research ethics approval	24	Plans for seeking research ethics committee/institutional review board (REC/IRB) approval	____10________
Protocol amendments	25	Plans for communicating important protocol modifications (eg, changes to eligibility criteria, outcomes, analyses) to relevant parties (eg, investigators, REC/IRBs, trial participants, trial registries, journals, regulators)	____10________

For peer review only - http://bmjopen.bmj.com/site/about/guidelines.xhtml

Consent or assent	26a	Who will obtain informed consent or assent from potential trial participants or authorised surrogates, and how (see Item 32)	______7____
	26b	Additional consent provisions for collection and use of participant data and biological specimens in ancillary studies, if applicable	____________
Confidentiality	27	How personal information about potential and enrolled participants will be collected, shared, and maintained in order to protect confidentiality before, during, and after the trial	_____5______
Declaration of interests	28	Financial and other competing interests for principal investigators for the overall trial and each study site	_______15_____
Access to data	29	Statement of who will have access to the final trial dataset, and disclosure of contractual agreements that limit such access for investigators	_____5_______
Ancillary and post-trial care	30	Provisions, if any, for ancillary and post-trial care, and for compensation to those who suffer harm from trial participation	______________
Dissemination policy	31a	Plans for investigators and sponsor to communicate trial results to participants, healthcare professionals, the public, and other relevant groups (eg, via publication, reporting in results databases, or other data sharing arrangements), including any publication restrictions	_____10______
	31b	Authorship eligibility guidelines and any intended use of professional writers	______________
	31c	Plans, if any, for granting public access to the full protocol, participant-level dataset, and statistical code	______________
**Appendices**			
Informed consent materials	32	Model consent form and other related documentation given to participants and authorised surrogates	______________
Biological specimens	33	Plans for collection, laboratory evaluation, and storage of biological specimens for genetic or molecular analysis in the current trial and for future use in ancillary studies, if applicable	______________

*It is strongly recommended that this checklist be read in conjunction with the SPIRIT 2013 Explanation & Elaboration for important clarification on the items. Amendments to the protocol should be tracked and dated. The SPIRIT checklist is copyrighted by the SPIRIT Group under the Creative Commons "Attribution-NonCommercial-NoDerivs 3.0 Unported" license.

For peer review only - http://bmjopen.bmj.com/site/about/guidelines.xhtml

BMJ Open

Randomized double-blind phase 3 clinical study testing impact of atorvastatin on prostate cancer progression after initiation of androgen deprivation therapy - study protocol

Journal:	*BMJ Open*
Manuscript ID	bmjopen-2021-050264.R1
Article Type:	Protocol
Date Submitted by the Author:	16-Dec-2021
Complete List of Authors:	Siltari, Aino; Tampere University, Faculty of Medicine and Health Technology; University of Helsinki, Faculty of Medicine, Pharmacology<br>Riikonen, Jarno; Tampere University Hospital, Department of Urology<br>Koskimäki, Juha; Tampere University Hospital, Department of Urology<br>Pakarainen, Tomi; Tampere University Hospital, Department of Urology<br>Ettala, Otto; University of Turku<br>Boström, Peter; University of Turku<br>Seikkula, Heikki; Central Finland Central Hospital<br>Kotsar, Andres; Tartu University Hospital<br>Tammela, Teuvo; University of Tampere<br>Helminen, Mika; Tampere University, Health Sciences<br>Raittinen, Paavo; Aalto University School of Science and Technology, Department of Mathematics and Systems Analysis<br>Lehtimäki, Terho; Tampere University, Department of Clinical Chemistry<br>Fode, Mikkel; Herlev Hospital<br>Østergren, Peter; Herlev and Gentofte University Hospital, Department of Urology<br>Borre, Michael; Aarhus Universitetshospital, Department of Urology<br>Rannikko, Antti; Helsinki University and Helsinki University Hospital, Department of Urology<br>Marttila, Timo; Seinäjoki Central Hospital, Department of Urology<br>Salonen, Arto; Kuopio University Hospital<br>Ronkainen, Hanna; Oulu University Hospital<br>Löffeler, Sven; Vestfold Hospital Trust, Section of Urology<br>Murtola, Teemu J ; Tampere University Hospital, Department of Urology
**Primary Subject Heading**:	Urology
Secondary Subject Heading:	Oncology
Keywords:	Prostate disease < UROLOGY, Clinical trials < THERAPEUTICS, Urological tumours < ONCOLOGY

Randomized double-blind phase 3 clinical study testing impact of atorvastatin on prostate cancer progression after initiation of androgen deprivation therapy - study protocol

Aino Siltari[1], Jarno Riikonen[2], Juha Koskimäki[2], Tomi Pakarainen[2], Otto Ettala[3], Peter Boström[3], Heikki Seikkula[4], Andres Kotsar[5], Teuvo LJ Tammela[1,2], Mika Helminen[7,8], Paavo V Raittinen[9], Terho Lehtimäki[1,10], Mikkel Fode[11], Peter Østergren[11], Michael Borre[12], Antti Rannikko[13], Timo Marttila[14], Arto Salonen[15], Hanna Ronkainen[16], Sven Löffeler[17], Teemu J Murtola[1,2*]

[1]Tampere University, Faculty of Medicine and Health Technology, Tampere, Finland, [2]TAYS Cancer Center, Department of Urology, Tampere, Finland, [3]Turku University Hospital and University of Turku, Turku, Finland, [4]Central Finland Central Hospital, Jyväskylä, Finland, [5]Tartu University Hospital, Department of Urology, Tartu, Estonia, [6]Fimlab laboratories, Department of Pathology, Tampere, Finland, [7]Tampere University Hospital, Research Services, Tampere, Finland, [8]Tampere University, Faculty of Social Sciences, Health Sciences, Tampere Finland, [9]Aalto University School of Science, Department of Mathematics and Systems Analysis, Espoo, Finland, [10]Fimlab Laboratories, Department of Clinical Chemistry, Tampere, Finland, [11]Department of Urology, Herlev and Gentofte Hospital, Denmark, [12]Aarhus University Hospital, Department of Urology, Aarhus, Denmark, [13]Helsinki University and Helsinki University Hospital, Department of Urology, Helsinki, Finland [14]Seinäjoki Central Hospital, Department of Urology, Seinäjoki, Finland, [15]Kuopio University Hospital, Department of Urology, Kuopio, Finland, [16]Oulu University Hospital, Department of Urology, Oulu, Finland, [17] Vestfold Hospital Trust, Section of Urology, Tønsberg, Norway

*corresponding author: Teemu J Murtola, Department of Urology, Tampere University Hospital, PL 2000, 33521 Tampere, Finland, teemu.murtola@tuni.fi, telephone: +358 (0) 294 52 11.

Keywords: Prostate cancer; castration-resistant prostate cancer; androgen deprivation therapy; atorvastatin; randomized clinical trial

Word count: 4069 (excluding title page, references, figures, and tables)

For peer review only

Abstract

Introduction

Blood cholesterol is likely a risk factor for prostate cancer prognosis and use of statins is associated with lowered risk of prostate cancer recurrence and progression. Furthermore, use of statins has been associated with prolonged time before development of castration resistance (CR) during androgen deprivation therapy (ADT) for prostate cancer. However, the efficacy of statins on delaying castration-resistance has not been tested in a randomized placebo-controlled setting.

This study aims to test statins' efficacy compared to placebo in delaying development of CR during ADT treatment for primary metastatic or recurrent prostate cancer. Secondary aim is to explore effect of statin intervention on prostate cancer mortality and lipid metabolism during ADT.

Methods and analysis

In this randomized placebo-controlled trial, a total of 400 men with *de novo* metastatic prostate cancer or recurrent disease after primary treatment and starting ADT will be recruited and randomized 1:1 to use daily 80 mg of atorvastatin or placebo. All researchers, study nurses, and patients will be blinded throughout the trial. Patients are followed until disease recurrence or death. Primary outcome is time to formation of CR after initiation of ADT. Serum lipid levels (total cholesterol, HDL, LDL, and trigyserides) are analyzed to test whether changes in serum cholesterol parameters during ADT predict length of treatment response. Furthermore, the trial will compare quality of life, cardiovascular morbidity, changes in blood glucose and circulating cell-free DNA, and urine lipidome during trial.

Ethics and dissemination

This study is approved by the Regional ethics committees of the Pirkanmaa Hospital District, Science center, Tampere, Finland (R18065M) and Tarto University Hospital, Tarto, Estonia (319/T-6). All participants read and sign informed consent form before study entry. Clinicaltrial.gov: NCT04026230, Eudra-CT: 2016-004774-17, protocol code: ESTO2, protocol date September 10th 2020 and version 6. After publication of results for the primary endpoints, anonymized summary metadata and statistical code will be made openly available. The data will not include any information that could make it possible to identify a given participant.

For peer review only - http://bmjopen.bmj.com/site/about/guidelines.xhtml

Keywords: Prostate cancer; castration-resistant prostate cancer; androgen deprivation therapy; atorvastin; randomized clinical trial

Strengths and limitations of this study

- First randomized placebo-controlled phase 3 clinical study on effects of atorvastatin on prostate cancer progression during ADT treatment
- Multicenter study in Finland, Denmark, Estonia, and Norway
- As a limiting factor, only minority of prostate cancer cases are advanced and large proportion of potential participants are ineligible due to using statins already. Thus, study enrolment will take several years

Introduction

Prostate cancer (PCa) is the most common cancer in Finnish men and a major public health burden causing annually around 900 cancer deaths in Finland and the yearly costs caused by the disease are estimated to reach 180 million euros.[1-2] However, not all prostatic malignancies are lethal; only 10-20% of tumors advance to metastases and eventually into a fatal stage. Advanced PCa is treated by androgen deprivation therapy (ADT). Eventually, however, PCa progress despite the ADT treatment and forms state called castration resistance. Thus, castration resistance is clinically defined as a moment when prostate cancer no longer responds to ADT treatment. Median time to castration resistance is 12-15 months and 45 months to death in patients who have started ADT for metastatic prostate cancer.[3-7] In men who start ADT for disease recurrence after primary treatment, median failure-free survival time is 33 months and 70 months to death.[3]

Blood cholesterol is likely a risk factor for prostate cancer prognosis; risk of disease recurrence after primary treatment is significantly elevated in men with hypercholesterolemia.[8] Laboratory studies have demonstrated the importance of cholesterol for prostate cancer cell growth.[9,10] Furthermore, upregulation of intracellular lipid production and ensuing lipid accumulation is essential in surviving hypoxic tumor microenvironment.[11] In prostate cancer cells it also assists in evading host-tumor immune response.[12] Also, upregulation of intracellular cholesterol production appears to be central for development of castration resistance.[13]

For peer review only

Lipid metabolism is emerging as a new risk factor for prostate cancer progression. The role of cholesterol is important in development of castration resistance, and inhibition of intracellular lipid production interferes with androgen receptor (AR) signaling essential for prostate cancer progression[13-15]. Cholesterol is precursor for intracellular androgen production, a central mechanism for prostate cancer cells to overcome ADT.[13,15] A recent study suggests that some of the serum basic lipid parameters may have increased association with development of castration resistance and metastasis especially in statin-naïve patients.[16] Clarification of the role of lipid metabolism during ADT will likely provide new tools for control of disease progression and prostate cancer treatment.

Use of cholesterol-lowering statin drugs is associated with lowered risk of prostate cancer recurrence and progression; risk of prostate cancer death is reduced by 30% compared to the non-users.[14,17,18] The anticancer effect may be specifically against progression of the disease and the mechanisms driving it.[19] Statins inhibit the cholesterol-synthesizing mevalonate pathway, which is active in prostate cancer cells.[10,20] Besides cholesterol, this pathway also produces isoprenoid proteins which are critical for regulation of cell growth and other central cellular control processes.[20] Furthermore, steroid hormones such as testosterone are metabolized from cholesterol, thus statins appear to target androgen metabolism, another crucial pathway for prostate cancer growth.[21,22]

Statin use has been reported to prolong efficacy of ADT in prostate cancer as it has been reported to prolong the efficacy of ADT for 8-10 months.[23,24] Furthermore, statins have been linked to a prolonged response to androgen-signaling targeted drugs abiraterone and enzalutamide used in management of castration-resistant disease.[25,26]

Statins have not been found to affect prostate cancer mortality in trials testing their efficacy in secondary prevention of cardiovascular disease.[27] However, in these trials cancer was often an exclusion criterion. On the other hand, it may be that statin treatment has more impact on hormone-dependent cancers which are underrepresented in these studies. In a randomized clinical trial focusing on prostate cancer patients 80 mg of atorvastatin has been found to reduce tumor proliferation activity compared to placebo after minimum exposure of 21 days.[28] Similar results were seen after treatment with fluvastatin, although in a non-randomized and uncontrolled setting.[29] Nevertheless, clinical efficacy of statins in preventing progression of prostate cancer has not been tested in a randomized placebo-controlled setting. Also, a recent post-hoc study of randomized clinical trial concluded that statin use was associated with decreased overall and PCa-specific mortality in men with ADT.[30] Therefore, it is important to do a trial testing effects of statins specifically in prostate cancer patients.

For peer review only

Study objective

Primary objective for this phase III randomized double-blind placebo-controlled trial is to explore whether intervention with atorvastatin delays prostate cancer progression i.e. development of castration resistance compared to placebo during androgen deprivation therapy (ADT) for metastatic or recurrent prostate cancer. Secondary objectives include exploring whether atorvastatin lowers prostate cancer-specific or overall mortality compared to placebo, and to demonstrate whether changes in serum lipid parameters predict disease recurrence and occurrence of adverse tumor genomic traits predicting castration resistance among prostate cancer patients during ADT.

Methods and analysis

Study setting

Study flow, study settings, and other information are presented in Figure 1 and Table 1. The study recruitment target is 400 participants who start ADT as primary management of *de novo* metastatic prostate cancer or as secondary management for prostate cancer recurrence after localize treatment. Participants can be high-risk M0 or M1 stage, main inclusion criterion is that long-term ADT treatment is started. These men will be randomized 1:1 (200 + 200) to receive either 80 mg of atorvastatin daily or placebo until disease recurrence i.e. development of castration resistance, death, or maximum of ten years. Sample size is based on a power calculation from a previous retrospective study.[23]

The study will be carried out in collaboration between urological departments of University Hospitals and central hospitals in Finland, the Herlev University Hospital in Denmark, the Tartu University Hospital in Estonia, and Vestfold Hospital Trust in Norway (Table 1).

Study data are collected and managed using REDCap electronic data capture tools hosted at Tampere University.[31,32] Only the primary investigators, study nurses, and registered study co-investigator from each participating site will have access to the platform. All laboratory results, symptoms, and results from imaging studies done at the discretion of the attending clinicians are recorded in the database. All clinical decisions besides the study drug, e.g. use of early chemotherapy, abiraterone, or other drugs in adjunct with ADT will be up to the discretion of the attending clinician and allowed but will also be recorded in the REDCap database.

Follow-up is continued until development of castration resistance, death, or maximum of 10 years. Participants are given the opportunity to carry on with the intervention even after development of

For peer review only - http://bmjopen.bmj.com/site/about/guidelines.xhtml

castration resistance to observe effects on survival. Unblinding will be performed after recruitment target has been reached and all participants have been followed for minimum of 12 months.

Castration resistance is defined as either PSA progression (three consecutive rises of PSA measured at least 1 week apart with two > 50% increases over the nadir and PSA > 2 ng/ml) or radiological progression (appearance of two or more lesions in bone scan or soft tissue enlargement as per RECIST criteria) while serum testosterone is at the castrate level (< 50 ng/ml or 1.7 nmol/l).

For men who initiate statin use during the study period for clinical indications, the study drug is dropped but the study follow-up is continued. These men will be included in the final analysis according to the intention-to-treat principle within their allocated study arm.

In case of intolerable side-effects as judged by either the participant or the attending physician, the study drug is stopped, and these men will be analyzed according to the intention-to-treat principle.

Participants who discontinue follow-up or deviate from the study protocol for any reason will be given the chance to remain or return to follow-up to allow intention-to-treat analyses.

Inclusion and exclusion criteria

Inclusion criteria for participants are histopathologically confirmed metastatic adenocarcinoma of the prostate or high-risk M0 stage recurrent prostate cancer for which androgen deprivation or antiandrogen therapy is initiated no longer than 3 months before recruitment, willingness to participate, and signing of informed consent (Figure 1).

Exclusion criteria for participants are regular statin use at the time of recruitment or within 6 months of it, previous adverse effects during statin therapy, familial hypercholesterolemia or very high total cholesterol (9.3 mmol/l or above), clinically significant renal (serum creatinine above 170 µmol/l) or liver insufficiency (serum alanine aminotransferase more than two times above the upper limit of normal range), and use of drugs that may interact with statins (St John's Wort, HIV protease inhibitors, ciclosporin, macrolide antibiotics, fucidic acid, phenytoin, carbamazepine, dronedarone, or oral antifungal medication) (Figure 1).

Study endpoints

Primary endpoint is the time to disease progression after starting ADT/antiandrogen therapy. Secondary endpoints are 1); prostate cancer-specific mortality and overall survival, 2); change in

For peer review only - http://bmjopen.bmj.com/site/about/guidelines.xhtml

serum cholesterol during the intervention and its role in predicting time to disease recurrence in the placebo arm, 3); occurrence of adverse tumor traits predicting development of castration resistance in circulating cell free DNA, 4); changes in fasting blood glucose during ADT, 5); occurrence of cardiovascular events during ADT, and 6); Quality of life (QoL) during ADT.

Study flow

Study flow and schedule is presented in Figure 1 and Table 2. Patients are recruited at urology outpatient clinic at urologists' visits. If the inclusion criteria are met and the exclusion criteria are not and the participant signs informed consent form, he is given a study number (ranging between E-001 to E-400) and he receives either 80 mg atorvastatin or placebo according to the study arm randomly allocated for the study number. No blocking or other restrictions will be implemented for randomization. Only the national study coordinator in each country will be able to see which study arm the participant has been randomized to. The participant will then receive first dose of the respective study drug randomized to his study number. The drug boxes and capsules containing atorvastatin and placebo will be identical in appearance. All researchers, nurses, and study participants will remain blinded to the allocation sequence until termination of the study and closure of the study data.

Control visits are scheduled at 6-month intervals to suit common clinical practice. Control visits are done by a clinician, complemented with measurements done by a research nurse. For each control visit the participant returns remaining capsules from the previously delivered ration and will receive a new one according to his study number. Number of the remaining capsules will be counted and saved in the REDCap, trial database, to monitor compliance. In every visit serum PSA (ng/ml), lipid levels (total cholesterol, HDL, LDL, and triglycerides, mmol/l), alkaline phosphatase (U/l), creatinine (µmol/l), and fasting glucose (mmol/l) will be measured (Table 2) and the participant is asked about symptoms that are suggestive of metastases and about possible adverse effects of the study drug. Serum creatine kinase (U/l) and alanine aminotransferase (U/l) is also measured at each visit.

Blood samples are taken and stored at 6-month intervals to monitor changes in tumor markers and occurrence of adverse tumor characteristics after initiation of ADT. Quality of life during the intervention will be charted once a year using validated WHOQOL-BREF quality of life questionnaire.[33]

Follow-up is continued until development of castration resistance, drop out from the study, death, or maximum of ten years. Participants developing castration resistance are given an opportunity to carry

For peer review only - http://bmjopen.bmj.com/site/about/guidelines.xhtml

on with their assigned treatment as statins have been linked to longer cancer-specific survival. Unblinding will be performed after recruitment target has been reached and all participants have been followed for minimum of 12 months.

Prostate cancer treatment apart from the study drug will be up to the discretion of the attending clinician. All laboratory results, symptoms, and possible imaging results will be recorded in the REDCap database. All clinical decisions regarding early chemotherapy and imaging will be made on the discretion of the clinician and recorded in the REDCap database.

Stored blood samples

Separate whole blood (for RNA and DNA isolation), plasma, and serum samples are taken at each control visit at 6-month intervals and stored for mass-spectrometric and nuclear magnetic resonance (NMR)-based determination of serum lipidome. These samples will also be used for RNA and whole genome sequencing of mutations and genetic modifications predicting disease recurrence and metastasis, such as BRCA1/2, ERG, MYC, TP53, ATM, PTEN, and AR splice variants.[34,35] Also hypoxia markers will be measured.[36]

For participants with confirmed metastases in the bone or soft tissues, amount of cell free DNA from the plasma will be measured before ADT initiation and again at castration resistance development for sequencing and detection of genetic modifications predicting metastases.[34,35] Selected patients are also imaged with positron emission tomography (PET) scan using 18F-2-nitroimidazolpentafluoropropylacetamide (EF5)[37] to monitor hypoxia and FDG-PET to evaluate immune responses[38] in the primary tumor and the metastases during the atorvastatin intervention.

Sample size calculation

In a cohort study by Harshman et al.[23], among men starting ADT, 58% of the statin users and 75% of non-users of statins progress to castration resistance during median 5.8 years. We used these crude percentages for sample size calculations.

With alpha and beta values of 0.05 and 0.20 (power=0.80), sample size of 400 men will be enough to detect a risk difference with HR 0.65 (Figure 2). We assume 10% drop-out rate in each study arm. The program "PS - Power and sample size, version 3.1.2" was used for the calculation.

The median time to disease progression is assumed to be 12-15 months for patients with *de novo* metastatic disease and 33 months for patients recurring after primary therapy.[3-7] Therefore, the

For peer review only - http://bmjopen.bmj.com/site/about/guidelines.xhtml

intervention will continue until castration resistance, death, or for maximum of 10 years. Post hoc follow-up will continue after the intervention. A 3 months' difference in time to castration resistance between the study arms will be considered clinically significant.

It has to be noted that the number of studies published on this topic is low and, in the absence of randomized evidence on this topic, the power calculation is based on results of epidemiological study prone to residual bias. Therefore, an interim analysis will be performed after the first 100 participants have met the primary endpoint, i.e. progressed into castration resistance. Without lifting the blinding, the power calculations are repeated to verify whether the chosen sample size has adequate statistical power to detect statistically significant difference of the effect size observed between the randomization groups at that time. If the calculations suggest that a larger sample size is needed for the trial, the study protocol will be amended with updated recruitment target.

Statistical methods

Distribution balance of prostate cancer clinical characteristics such as TNM stage at diagnosis, Gleason score, and PSA at time of diagnosis will be presented in a patient characteristics table, stratified by study arms. Categorical variables are displayed as absolute count frequencies and relative frequencies, i.e., proportion within study arm. Continuous variables are presented as median and interquartile range. Minimum and maximum values are not shown to ensure anonymity of the participants.

Recurrence-free survival, i.e. time from ADT initiation to castration resistance, will be analyzed by using Kaplan-Meier (KM) estimator, stratified by study arm. The statistical significance of the difference between the survival estimates is tested with log-rank test. No adjustments will be used for KM estimator as the study intervention is randomized. Risk of death, as well as risk for occurrence of adverse tumor traits among men receiving atorvastatin compared to men receiving placebo will be estimated using Cox proportional hazards regression.

Univariate analysis of changes in serum lipid levels is performed by calculating the difference after – before for each participant. The statistical significance of the difference between the study arms is analyzed by Mann-Whitney U-test (or by t-test if under normality). These results are complemented with boxplots. Multivariable analysis for difference in serum lipid levels between the study arms are performed by fitting a logistic regression model such that the study arm is used as the response variable whereas lipid level differences are used as predictors (i.e., each lipid level difference as an independent predictor). Lipid levels are expected to display heavy multi-collinearity; therefore,

For peer review only

For peer review only - http://bmjopen.bmj.com/site/about/guidelines.xhtml

variance inflation factors (VIF) are calculated for each coefficient afterwards, and possible adjustments made by dropping predictors with extreme VIFs (> 10).

The occurrence of adverse tumor genomic traits will be analysed by calculating the proportion of participants developing adverse tumor genomic traits. The statistical significance between the proportions is tested by proportions test.

The WHOQOL-BREF patient reported outcome (PRO) of the quality-of-life is analysed by tabulating absolute and relative frequencies. Chi-squared test for homogeneity is used to test the statistical significance of the difference between the study arms of each PRO item

Subgroup analyses will be performed stratified by type of ADT or antiandrogen therapy initially selected; GnRH agonists/antagonists, antiandrogen, orchiectomy, or other. Men who start with antiandrogen therapy often change to GnRH agonists/antagonists or orchiectomy when PSA increases during ADT. This is allowed and PSA recurrence during antiandrogen therapy is not considered as disease progression as serum testosterone needs to be at castrate level as well for meeting the primary endpoint.

Another subgroup analysis will be stratified by whether or not the participants have received local therapy (radiation or surgery) for prostate cancer in addition to ADT and by type of additional therapy participant has received; docetaxel, abiraterone, enzalutamide, or apalutamide at hormone-sensitive stage in addition to ADT.

All analyses will include only participants with available data, imputations will not be used. Detailed statistical analysis plan is published before final data analysis. All analyses are performed using SPSS, Stata and R statistical softwares.

Quality control

The study steering committee includes investigators from each participating recruitment center. The steering committee will meet twice a year to oversee trial progression of the recruitment, integrity of collected data and discuss possible protocol amendments.

External trained study monitors independent from the study sponsors ensure the data quality and good clinical practice by making regular yearly check-up visits to participating study centers. This will be separately arranged in each participating country.

For peer review only - http://bmjopen.bmj.com/site/about/guidelines.xhtml

Reporting and registering of adverse effects

The participants are advised to contact the researchers in case of suspected adverse effect related to the study drug. If the side-effects are intolerable the study drug will be discontinued. In uncertain cases serum levels of creatine kinase (U/l), alanine aminotranspherase (U/l) and creatinine (µmol/l) are checked to see if any of these have changed considerably compared to the baseline. If the laboratory tests are not normal, or if the participant or the attending physician finds the side-effects alarming the study drug will be discontinued. Study database records specifically most common adverse effects muscular pain, elevated fasting blood glucose (> 6,1 mmol/l) and elevated serum creatine kinase (>280 U/l), even when they do not lead to discontinuation of the study drug.

Trial database, REDCap, includes separate question for serious adverse effects as evaluated by CTCAE criteria, version 5.0. If serious side-effects are reported, REDCap asks to fulfill separate query where detailed description of the adverse effect is given according to CTCAE criteria. Study sponsor and coordinator will be automatically notified via e-mail if serious adverse effect is reported in the database. All serious adverse effect that threatens life or health are reported to national authority (FIMEA in Finland) within 7 days of detection. If a life-threatening adverse effect is suspected blinding will be lifted for the participant in case to see whether he has received atorvastatin or placebo. Unexpected adverse effects will be reported to national authority within 15 days of detection.

Patients and public involvement

In the design phase of the trial, primary investigator presented the study protocol in numerous public events, including patient advocacy group meetings, giving opportunity for the audience to give feedback and suggestions how to improve the study. Patient advocacy organizations of prostate cancer patients are actively involved in finding subjects for the study through announcements in their journals and by allowing investigators frequently promote the study in their meetings. Study results will be also reported in patient organization journals and social media sites for maximum visibility and distribution of trial outcomes.

Ethics and dissemination

This study is approved by the regional ethics committee of Pirkanmaa Hospital District, Science center, Tampere, Finland (R18065M). All participants read and sign informed consent form before study entry. The results of this study will be published in international peer reviewed journals.

For peer review only - http://bmjopen.bmj.com/site/about/guidelines.xhtml

The trial is ethical for the following reasons 1); The study aims to improve treatment of metastatic prostate cancer, which is the second most common cause of cancer death in Western countries, 2); the potential scientific and societal benefits from the project are substantial. If the study hypothesis proves to be right, it will provide an entirely new way to prevent and/or delay progression of metastatic prostate cancer with atorvastatin, a drug that is cheap, well-tolerated, and with established cardiovascular benefits, 3); the study drug (atorvastatin) is known to be well-tolerated and it is currently widely used in management of hypercholesterolemia and cardiovascular disease. The adverse effects caused by the drug are usually mild and transient after stopping the study drug, and 4); randomized, placebo-controlled design ensures the study will produce highest quality evidence that will change prostate cancer treatment guidelines if the hypothesis proves to be right.

After publication of results for the primary endpoints, anonymized summary metadata and statistical code will be made openly available. The data will not include any information that could make it possible to identify a given participant.

References

1. Finnish cancer registry statistics: http://stats.cancerregistry.fi/stats/eng/veng0003m0.html
2. Mäklin S, Rissanen P. Cancer expenses in Finland: http://www.cancer.fi/@Bin/47734/Syöpien+aiheuttamat+kustannukset.pdf
3. Ross RW Xie W, Regan MM, Pomerantz M, *et al*. Efficacy of androgen deprivation therapy (ADT) in patients with advanced prostate cancer: association between Gleason score, prostate-specific antigen level, and prior ADT exposure with duration of ADT effect. *Cancer* 2008;**112**:1247-53. doi: 10.1002/cncr.23304.
4. Botrel TE, Clark O, Lima Pompeo AC, *et al*. Efficacy and Safety of Combined Androgen Deprivation Therapy (ADT) and Docetaxel Compared with ADT Alone for Metastatic Hormone-Naive Prostate Cancer: A Systematic Review and Meta-Analysis. *PLoS One* 2016;**11**:e0157660. doi: 10.1371/journal.pone.0157660.
5. Sweeney CJ, Chen YH, Carducci M, *et al.* Chemohormonal Therapy in Metastatic Hormone-Sensitive Prostate Cancer. *N Engl J Med* 2015;**373**:737-46. doi: 10.1056/NEJMoa1503747
6. Seo WI, Kang PM, Kim TH, *et al*. Primary androgen deprivation therapy for prostate cancer in koreans: a retrospective multicenter study. World J Mens Health. 2014;32(3):159-66.

For peer review only

7. Mottet N, Van Damme J, Loulidi S, Russel C, Leitenberger A, Wolff JM. Intermittent hormonal therapy in the treatment of metastatic prostate cancer: a randomized trial. *BJU Int* 2012;**110**:1262-9. doi: 10.1111/j.1464-410X.2012.11120.x.

8. Arthur R, Møller H, Garmo H*, et al.* Association between baseline serum glucose, triglycerides and total cholesterol, and prostate cancer risk categories. *Cancer Med* 2016;**5**:1307-18. doi: 10.1002/cam4.665

9. Murtola TJ, Syvälä H, Pennanen P, *et al.* Comparative effects of high and low-dose simvastatin on prostate epithelial cells: the role of LDL. *Eur J Pharmacol* 2011;**673**:96-100. doi: 10.1016/j.ejphar.2011.10.022.

10. Murtola TJ, Syvälä H, Pennanen P, *et al.* The importance of LDL and cholesterol metabolism for prostate epithelial cell growth. *PLoS One* 2012;**7**:e39445. doi: 10.1371/journal.pone.0039445.

11. Ackerman D, Simon MC. Hypoxia, lipids, and cancer: surviving the harsh tumor microenvironment. *Trends Cell Biol* 2014;**24**:472-8. doi: 10.1016/j.tcb.2014.06.001.

12. Herber DL, Cao W, Nefedova Y *et al*. Lipid accumulation and dendritic cell dysfunction in cancer. *Nat Med* 2010;**16**:880-6. doi: 10.1038/nm.2172

13. Han W, Gao S, Barrett D, *et al*. Reactivation of androgen receptor-regulated lipid biosynthesis drives the progression of castration-resistant prostate cancer. *Oncogene* 2018;**37**:710-21. doi: 10.1038/onc.2017.385.

14. Raval AD, Thakker D, Negi H, *et al.* Association between statins and clinical outcomes among men with prostate cancer: a systematic review and meta-analysis. *Prostate Cancer Prostatic Dis* 2016;**19**:222. doi: 10.1038/pcan.2016.3

15. Barfeld SJ, Itkonen HM, Urbanucci A, *et a*l. Androgen-regulated metabolism and biosynthesis in prostate cancer. *Endocr Relat Cancer* 2014;**21**:T57-66. doi: 10.1530/ERC-13-0515.

16. Dambal S, Howard LE, Allott EH, *et al*. Serum Lipids prior to Starting Androgen Deprivation Therapy and Risk of Castration Resistant Prostate Cancer and Metastasis: Results from the SEARCH Database. *J Urol* 2020;**203**:120-7. doi: 10.1097/JU.0000000000000494

17. Van Rompay MI, Solomon KR, Nickel JC, *et al*. Prostate cancer incidence and mortality among men using statins and non-statin lipid-lowering medications. *Eur J Cancer* 2019;**112**:118-26. doi: 10.1016/j.ejca.2018.11.033.

18. Kumar A, Riviere P, Luterstein E, *et al*. Associations among statins, preventive care, and prostate cancer mortality. *Prostate Cancer Prostatic Dis* 2020. doi: 10.1038/s41391-020-0207-5

For peer review only

19. Murai T. Cholesterol lowering: role in cancer prevention and treatment. *Biol Chem* 2015;**396**:1-11. doi: 10.1515/hsz-2014-0194.
20. Garcia-Ruiz C, Morales A, Fernandez-Checa JC. Statins and protein prenylation in cancer cell biology and therapy. *Anticancer Agents Med Chem* 2012;**12**:303-15. doi: 10.2174/187152012800228715
21. Armandari I, Hamid AR, Verhaegh G, *et al*. Intratumoral steroidogenesis in castration-resistant prostate cancer: a target for therapy. *Prostate Int* 2014;**2**:105-13. doi: 10.12954/PI.14063.
22. Mokarram P, Alizadeh J, Razban V, *et al*. Interconnection of Estrogen/Testosterone Metabolism and Mevalonate Pathway in Breast and Prostate Cancers. *Curr Mol Pharmacol* 2017;**10**:86-114. doi: 10.2174/1874467209666160112125631.
23. Harshman LC, Wang X, Nakabayashi M, et al. Statin use at the time of initiation of androgen deprivation therapy and time to progression in patients with hormone-sensitive prostate cancer. *JAMA Oncol* 2015;**1**:495-504. doi: 10.1001/jamaoncol.2015.0829.
24. Jung J, Lee C, Lee C, et al. Effects of statin use on the response duration to androgen deprivation therapy in metastatic prostate cancer. *Korean J Uro*l 2015;**56**:630-6. doi: 10.4111/kju.2015.56.9.630.
25. Syvälä H, Pennanen P, Bläuer M, *et al*. Additive inhibitory effects of simvastatin and enzalutamide on androgen-sensitive LNCaP and VCaP prostate cancer cells. *Biochem Biophys Res Commun* 2016;**481**:46-50. doi: 10.1016/j.bbrc.2016.11.021
26. Yang H, Pang L, Hu X, *et al.* The effect of statins on advanced prostate cancer patients with androgen deprivation therapy or abiraterone/enzalutamide: A systematic review and meta-analysis. *J Clin Pharm Ther* 2020;**45**:488-95. doi: 10.1111/jcpt.13092
27. Lv HL, Jin DM, Liu M, *et al*. Long-term efficacy and safety of statin treatment beyond six years: a meta-analysis of randomized controlled trials with extended follow-up. *Pharmacol Res* 2014;**81**:64-73. doi: 10.1016/j.phrs.2014.02.006.
28. Murtola T, Riikonen J, Syvälä H, *et al*. Atorvastatin Versus Placebo for Prostate Cancer Before Radical Prostatectomy-A Randomized, Double-blind, Placebo-controlled Clinical Trial. *Eur Urol* 2018;**74**:697-701. doi: 10.1016/j.eururo.2018.06.037.
29. Longo J, Hamilton RJ, Masoomian M, *et al.* A pilot window-of-opportunity study of preoperative fluvastatin in localized prostate cancer. *Prostate Cancer Prostatic Dis* 2020 ;**23**:630-37. doi: 10.1038/s41391-020-0221-7

For peer review only - http://bmjopen.bmj.com/site/about/guidelines.xhtml

30. Hamilton RJ, Ding K, Crook JM, *et al.* The Association Between Statin Use and Outcomes in Patients Initiating Androgen Deprivation Therapy. *Eur Urol* 2020 (article in press) https://doi.org/10.1016/j.eururo.2020.12.031
31. Harris PA, Taylor R, Thielke R, *et al.* Research electronic data capture (REDCap) – A metadata-driven methodology and workflow process for providing translational research informatics support. *J Biomed Inform* 2009;**42**:377-81. doi: 10.1016/j.jbi.2008.08.010
32. Harris PA, Taylor R, Minor BL, *et al*. The REDCap consortium: Building an international community of software partners. *J Biomed Inform* 2019;**95**:103208. doi: 10.1016/j.jbi.2019.103208.
33. WHOQOL-BREF, The World Health Organization Quality of Life, THL
34. Mateo J, Boysen G, Barbieri CE, *et al*. DNA repair in prostate cancer: biology and clinical implications. *Eur Urol* 2017;**71**:417-25. doi: 10.1016/j.eururo.2016.08.037
35. Xu J, Qiu Y. Role of androgen receptor splice variants in prostate cancer metastasis. *Asian J Urol* 2016;**3**:177-84. doi: 10.1016/j.ajur.2016.08.003.
36. Le QT, Courter D. Clinical biomarkers for hypoxia targeting. *Cancer Metastasis Rev* 2008;**27**:351-62. doi: 10.1007/s10555-008-9144-9
37. Stieb S, Eleftheriou A, Warnock G, *et al*. Longitudinal PET imaging of tumor hypoxia during the course of radiotherapy. *Eur J Nucl Med Mol Imaging* 2018;**45**:2201-17.doi: 10.1007/s00259-018-4116-y
38. Hunt SJ, Seraj SM, Alavi A. PET Assessment of Immune Effects from Interventional Oncology Procedures. *PET Clin* 2019;**14**:477-85. doi: 10.1016/j.cpet.2019.06.007.

Authors' contributions: T.J.M. is the primarily investigator of this study and designed and planned the study with the help from all other authors. J.R., T.P., O.E., P.B., H.S., A.K., T.L.J.T., M.F., P.Ø., A.R., T.M., A.Sa., H.R., S.L., and T.J.M. will be conducting the study at the study centers. M.H. and P.V.R. planned and will conduct the statistical analysis of the study. T.L helped to plan and collaborate in analysis of lipidomic profile. A.Si is coordinator of the trial. A.Si. and T.J.M. wrote the first draft of the manuscript. All other authors further contributed to manuscript preparation and accepted the final version of the manuscript.

Funding: This study is supported by grants from Finnish Cancer Foundation (grant numbers 024194 and 3122800563 (TJM) and 16112016 (TL)), Nordic Cancer Union (grant number MS793 (TJM)), Päivikki and Sakari Sohlberg Foundation (grant number 31228005901 (AS)), competitive research grant of Pirkanmaa hospital district (grant number 9X032 (TJM) and grant X51001 (TL)), and the Academy of Finland (grants numbers 322098 (TL), and 3121330724 (TJM)).

For peer review only - http://bmjopen.bmj.com/site/about/guidelines.xhtml

Disclaimer: None of the funders has a role in the design, management, analysis or interpretation of data, or writing and publication of results.

Competing interests: Peter Østergren: honorarium as speaker from Ipsen A/S, Ferring Pharmaceuticals, and Astellas Pharma. Mikkel Fode: consultant fees and honorarium as speaker from Astellas and Ferring. Hanna Ronkainen: consultant fees from Bayer AB and honorarium as speaker from Sanofi. Teemu J Murtola: Consultant fees from Astellas, Janssen, speaker's honorarium from Astellas, Janssen and Sanofi, participation in scientific meetings at the expense of Ferring, Pfizer, and Sanofi, stockholder for Arocell AB. Peter Boström: consultant fees from Astellas Pharma. All other authors: No competing interests to declare.

Data sharing: There are no additional unpublished data at this point of this study.

Captions to Figure and Tables:

Figure 1. Study flow with inclusion and exclusion criteria.

Figure 2. Sample size estimation with power 0.8 as a function of hazard ratio. Calculation was made based on study by Harshman et al.[23]

Table 1. Study setting of Impact of atorvastatin on prostate cancer after initiation of androgen deprivation therapy -clinical trial

Table 2. Study flow and timing of the laboratory and blood sample collection

For peer review only

Table 1. Study setting of Impact of atorvastatin on prostate cancer after initiation of androgen deprivation therapy -clinical trial.

For peer review only

**Study settings**	
Primary registry and trial identifying number	Clinicaltrials.gov NCT04026230
Date of registration in primary registry	July 19, 2019
Secondary identifying numbers	Eudra-CT: 2016-004774-17, Protocol code: ESTO2
Source(s) of monetary or material support	Tampere University Hospital, Finland
Primary sponsor	Tampere University Hospital, Finland
Secondary sponsor(s)	Helsinki University Hospital,Turku University Hospital, Central Fi Central Hospital, Kuopio University Hospital, Oulu University Hos Finland, Herlev Hospital, Denmark, University Hospital Tarto, Est Vestfold Hospital Trust, Tønsberg, Norway
Contact for public queries	Tampere University Hospital, Teemu Murtola, MD, PhD
Contact for scientific queries	Tampere University Hospital, Teemu Murtola, MD, PhD
Public title	Impact of atorvastatin on prostate cancer progression after initi of androgen deprivation therapy
Scientific title	Impact of atorvastatin on prostate cancer progression after initi of androgen deprivation therapy – lipid metabolism as a novel biomarker to predict prostate cancer progression – phase 3, dou blind randomized clinical trial FinnProstata XV
Countries of recruitment	Finland, Denmark, Estonia, Norway
Health condition studied	Metastatic or recurrent prostate cancer
Intervention	Active comparator: Capsules of atorvastatin 80 mg, Placebo comparator: Similar capsules as in the atorvastatin arm, but wit the active ingredient
Key inclusion and exclusion criteria	Inclusion criteria: Histopathologically confirmed metastatic adenocarcinoma of the prostate for which androgen deprivation antiandrogen therapy is initiated no longer than 3 months befor the primary treatment<br>Willingness to participate and signing of informed consent<br>Exclusion criteria: Statin use at the time of recruitment or withi months of it, Previous adverse effects during statin therapy, fam hypercholesterolemia or very high total cholesterol ,clinically significant renal or liver insufficiency, use of drugs that may inte with statins
Sexes eligible for study:	Male
Accepts healthy volunteers	No
Study type	Interventional, Allocation: randomized, Intervention model: para assignment with 1:1 allocation ratio, Masking: double blind (sub caregiver, investigator, outcomes assessor), Primary purpose: prevention, Phase III
Date of first enrolment	July 2019
Target sample size	400
Recruitment status	Recruiting
Primary outcome(s)	Castration resistance
Key secondary outcomes	Lipid levels, Prostate cancer mortality and overall survival, Circu [illegible]

For peer review only

Table 2. Study flow and timing of the laboratory and blood sample collection.

	Enrollment	Allocation	Follow-up			
TIMEPOINT		0	6m	12m	18m	24m
ENROLLMENT:						
Eligibility screen	x					
Informed consent	x					
Allocation		x				
INTERVENTIONS:+						
Atorvastatin			x	x	x	x
Placebo			x	x	x	x
ASSESMENTS:						
Serum PSA (ng/ml)	x		x	x	x	x
Alkaline phosphatase (U/l)	x		x	x	x	x
Creatinine (µmol/l)	x		x	x	x	x
Serum creatine kinase (U/l)	x		x	x	x	x
Alanine aminotranspherase (U/l)	x		x	x	x	x
Fasting blood glucose (mmol/l)	x		x	x	x	x
Lipid levels (total cholesterol, LDL, HDL, and triglycerides) (mmol/l)	x		x	x	x	x
Cell free DNA (subgroup)^	x					
QoL	x			x		x

m=month, QoL=quality of life, PSA=prostate-specific antigen

*follow-up until disease recurrance, death, or max. 10 years

+ allocation 1:1 to have eighter 80 mg atorvastatin or placebo daily

^Cell free DNA is measured before allocation and after occurrence of castration resistance.

For peer review only

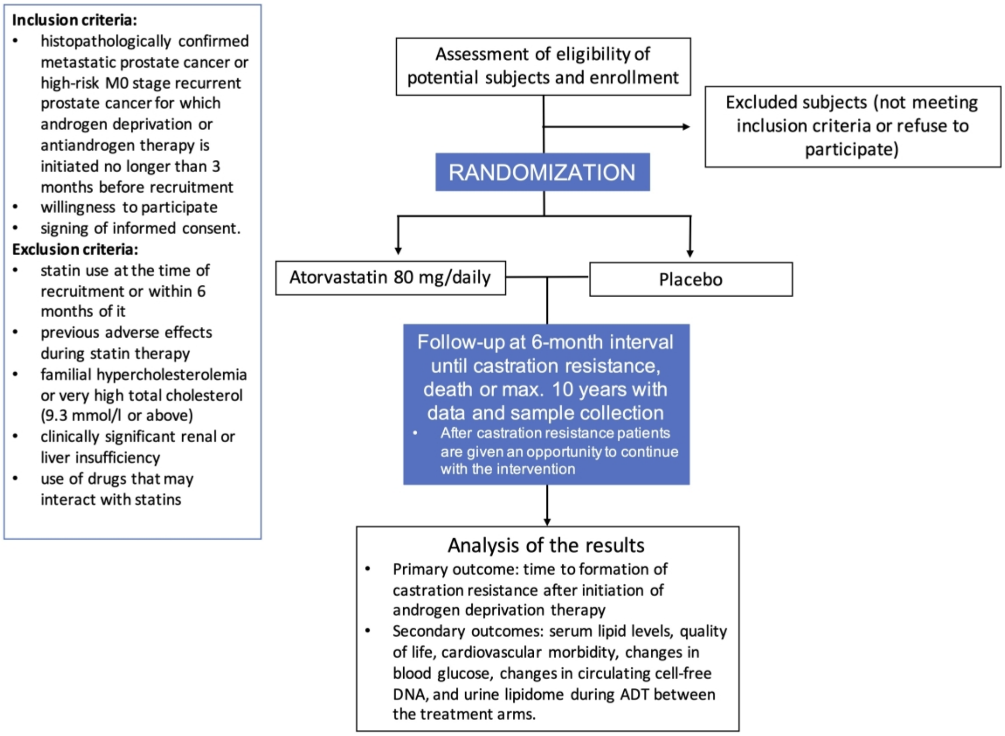

Study flow with inclusion and exclusion criteria.

1184x868mm (72 x 72 DPI)

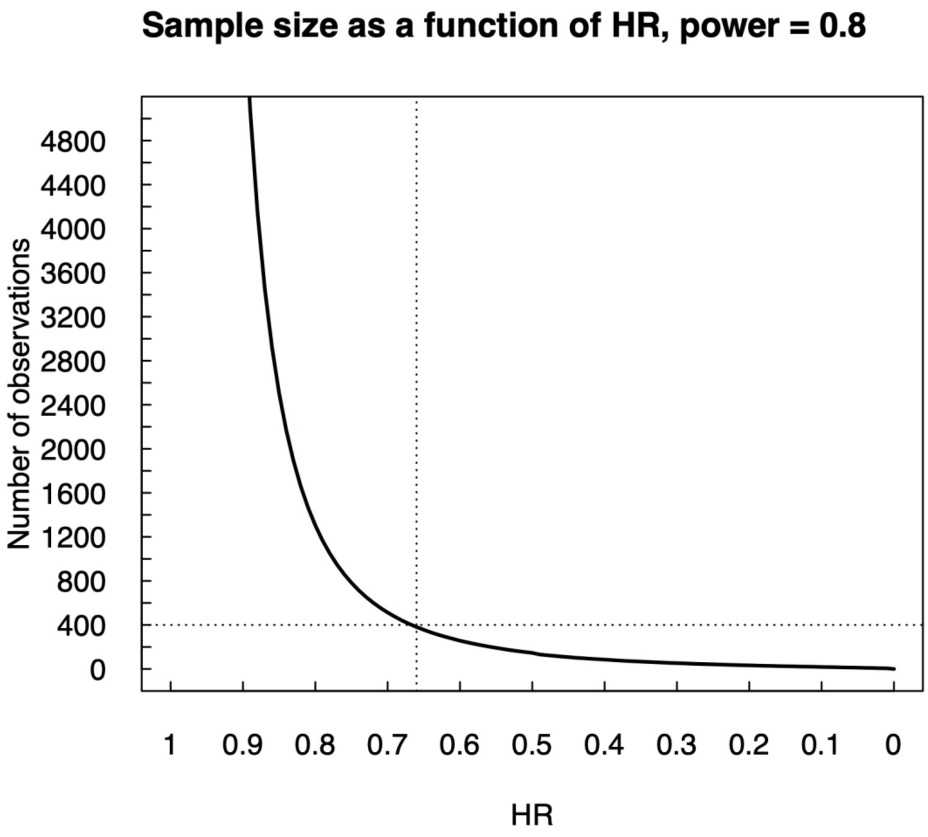

Sample size estimation with power 0.8 as a function of hazard ratio. Calculation was made based on study by Harshman et al.23

599x549mm (72 x 72 DPI)

SPIRIT

STANDARD PROTOCOL ITEMS: RECOMMENDATIONS FOR INTERVENTIONAL TRIALS

SPIRIT 2013 Checklist: Recommended items to address in a clinical trial protocol and related documents*

Section/item	Item No	Description	Addressed on page number
**Administrative information**			
Title	1	Descriptive title identifying the study design, population, interventions, and, if applicable, trial acronym	_______1______
Trial registration	2a	Trial identifier and registry name. If not yet registered, name of intended registry	_______2______
	2b	All items from the World Health Organization Trial Registration Data Set	______________
Protocol version	3	Date and version identifier	_________2_____
Funding	4	Sources and types of financial, material, and other support	______14_____
Roles and responsibilities	5a	Names, affiliations, and roles of protocol contributors	______1_______
	5b	Name and contact information for the trial sponsor	_______1______
	5c	Role of study sponsor and funders, if any, in study design; collection, management, analysis, and interpretation of data; writing of the report; and the decision to submit the report for publication, including whether they will have ultimate authority over any of these activities	______14_______
	5d	Composition, roles, and responsibilities of the coordinating centre, steering committee, endpoint adjudication committee, data management team, and other individuals or groups overseeing the trial, if applicable (see Item 21a for data monitoring committee)	______10_______

For peer review only

**Introduction**			
Background and rationale	6a	Description of research question and justification for undertaking the trial, including summary of relevant studies (published and unpublished) examining benefits and harms for each intervention	___3-4_______
	6b	Explanation for choice of comparators	___3-4_________
Objectives	7	Specific objectives or hypotheses	____5_____
Trial design	8	Description of trial design including type of trial (eg, parallel group, crossover, factorial, single group), allocation ratio, and framework (eg, superiority, equivalence, noninferiority, exploratory)	___5-6_____
**Methods: Participants, interventions, and outcomes**			
Study setting	9	Description of study settings (eg, community clinic, academic hospital) and list of countries where data will be collected. Reference to where list of study sites can be obtained	___5-6_________
Eligibility criteria	10	Inclusion and exclusion criteria for participants. If applicable, eligibility criteria for study centres and individuals who will perform the interventions (eg, surgeons, psychotherapists)	_____6_______
Interventions	11a	Interventions for each group with sufficient detail to allow replication, including how and when they will be administered	____7-8_______
	11b	Criteria for discontinuing or modifying allocated interventions for a given trial participant (eg, drug dose change in response to harms, participant request, or improving/worsening disease)	_______6______
	11c	Strategies to improve adherence to intervention protocols, and any procedures for monitoring adherence (eg, drug tablet return, laboratory tests)	____7-8________
	11d	Relevant concomitant care and interventions that are permitted or prohibited during the trial	______8______
Outcomes	12	Primary, secondary, and other outcomes, including the specific measurement variable (eg, systolic blood pressure), analysis metric (eg, change from baseline, final value, time to event), method of aggregation (eg, median, proportion), and time point for each outcome. Explanation of the clinical relevance of chosen efficacy and harm outcomes is strongly recommended	_______6-7____
Participant timeline	13	Time schedule of enrolment, interventions (including any run-ins and washouts), assessments, and visits for participants. A schematic diagram is highly recommended (see Figure)	_7-8,Table 2_____

For peer review only - http://bmjopen.bmj.com/site/about/guidelines.xhtml

Sample size	14	Estimated number of participants needed to achieve study objectives and how it was determined, including clinical and statistical assumptions supporting any sample size calculations	_____8-9______
Recruitment	15	Strategies for achieving adequate participant enrolment to reach target sample size	_____8-9_______
**Methods: Assignment of interventions (for controlled trials)**			
Allocation:			
Sequence generation	16a	Method of generating the allocation sequence (eg, computer-generated random numbers), and list of any factors for stratification. To reduce predictability of a random sequence, details of any planned restriction (eg, blocking) should be provided in a separate document that is unavailable to those who enrol participants or assign interventions	________7____
Allocation concealment mechanism	16b	Mechanism of implementing the allocation sequence (eg, central telephone; sequentially numbered, opaque, sealed envelopes), describing any steps to conceal the sequence until interventions are assigned	______7_____
Implementation	16c	Who will generate the allocation sequence, who will enrol participants, and who will assign participants to interventions	____7________
Blinding (masking)	17a	Who will be blinded after assignment to interventions (eg, trial participants, care providers, outcome assessors, data analysts), and how	______7_____
	17b	If blinded, circumstances under which unblinding is permissible, and procedure for revealing a participant's allocated intervention during the trial	_____7_______
**Methods: Data collection, management, and analysis**			
Data collection methods	18a	Plans for assessment and collection of outcome, baseline, and other trial data, including any related processes to promote data quality (eg, duplicate measurements, training of assessors) and a description of study instruments (eg, questionnaires, laboratory tests) along with their reliability and validity, if known. Reference to where data collection forms can be found, if not in the protocol	______7-8____
	18b	Plans to promote participant retention and complete follow-up, including list of any outcome data to be collected for participants who discontinue or deviate from intervention protocols	_____6______

Data management	19	Plans for data entry, coding, security, and storage, including any related processes to promote data quality (eg, double data entry; range checks for data values). Reference to where details of data management procedures can be found, if not in the protocol	________5_____
Statistical methods	20a	Statistical methods for analysing primary and secondary outcomes. Reference to where other details of the statistical analysis plan can be found, if not in the protocol	__9-10_________
	20b	Methods for any additional analyses (eg, subgroup and adjusted analyses)	_9-10_________
	20c	Definition of analysis population relating to protocol non-adherence (eg, as randomised analysis), and any statistical methods to handle missing data (eg, multiple imputation)	___10_______
**Methods: Monitoring**			
Data monitoring	21a	Composition of data monitoring committee (DMC); summary of its role and reporting structure; statement of whether it is independent from the sponsor and competing interests; and reference to where further details about its charter can be found, if not in the protocol. Alternatively, an explanation of why a DMC is not needed	_____10______
	21b	Description of any interim analyses and stopping guidelines, including who will have access to these interim results and make the final decision to terminate the trial	____6, 11______
Harms	22	Plans for collecting, assessing, reporting, and managing solicited and spontaneously reported adverse events and other unintended effects of trial interventions or trial conduct	______11_______
Auditing	23	Frequency and procedures for auditing trial conduct, if any, and whether the process will be independent from investigators and the sponsor	______10______
**Ethics and dissemination**			
Research ethics approval	24	Plans for seeking research ethics committee/institutional review board (REC/IRB) approval	____10________
Protocol amendments	25	Plans for communicating important protocol modifications (eg, changes to eligibility criteria, outcomes, analyses) to relevant parties (eg, investigators, REC/IRBs, trial participants, trial registries, journals, regulators)	____10________

Consent or assent	26a	Who will obtain informed consent or assent from potential trial participants or authorised surrogates, and how (see Item 32)	_______7____
	26b	Additional consent provisions for collection and use of participant data and biological specimens in ancillary studies, if applicable	____________
Confidentiality	27	How personal information about potential and enrolled participants will be collected, shared, and maintained in order to protect confidentiality before, during, and after the trial	_____5______
Declaration of interests	28	Financial and other competing interests for principal investigators for the overall trial and each study site	_______15_____
Access to data	29	Statement of who will have access to the final trial dataset, and disclosure of contractual agreements that limit such access for investigators	_____5_______
Ancillary and post-trial care	30	Provisions, if any, for ancillary and post-trial care, and for compensation to those who suffer harm from trial participation	______________
Dissemination policy	31a	Plans for investigators and sponsor to communicate trial results to participants, healthcare professionals, the public, and other relevant groups (eg, via publication, reporting in results databases, or other data sharing arrangements), including any publication restrictions	_____10______
	31b	Authorship eligibility guidelines and any intended use of professional writers	______________
	31c	Plans, if any, for granting public access to the full protocol, participant-level dataset, and statistical code	______________
**Appendices**			
Informed consent materials	32	Model consent form and other related documentation given to participants and authorised surrogates	______________
Biological specimens	33	Plans for collection, laboratory evaluation, and storage of biological specimens for genetic or molecular analysis in the current trial and for future use in ancillary studies, if applicable	______________

*It is strongly recommended that this checklist be read in conjunction with the SPIRIT 2013 Explanation & Elaboration for important clarification on the items. Amendments to the protocol should be tracked and dated. The SPIRIT checklist is copyrighted by the SPIRIT Group under the Creative Commons "Attribution-NonCommercial-NoDerivs 3.0 Unported" license.

For peer review only - http://bmjopen.bmj.com/site/about/guidelines.xhtml
